# *dldh^cri3^* zebrafish exhibit altered mitochondrial ultrastructure, morphology, and dysfunction partially rescued by probucol or thiamine

**DOI:** 10.1172/jci.insight.178973

**Published:** 2024-08-20

**Authors:** Manuela Lavorato, Donna Iadarola, Cristina Remes, Prabhjot Kaur, Chynna Broxton, Neal D. Mathew, Rui Xiao, Christoph Seiler, Eiko Nakamaru-Ogiso, Vernon E. Anderson, Marni J. Falk

**Affiliations:** 1 Mitochondrial Medicine Frontier Program, Division of Human Genetics, Department of Pediatrics, The Children’s Hospital of Philadelphia (CHOP), Philadelphia, Pennsylvania, USA.; 2 Department of Pediatrics and; 3 Department of Biostatistics, Epidemiology and Informatics, Perelman School of Medicine, University of Pennsylvania, Philadelphia, Pennsylvania, USA.; 4 Zebrafish Core, CHOP, Philadelphia, Pennsylvania, USA.

**Keywords:** Genetics, Metabolism, Drug therapy, Intermediary metabolism, Mitochondria

## Abstract

Dihydrolipoamide dehydrogenase (DLD) deficiency is a recessive mitochondrial disease caused by variants in *DLD*, the E_3_ subunit of mitochondrial α-keto (or 2-oxo) acid dehydrogenase complexes. DLD disease symptoms are multisystemic, variably manifesting as Leigh syndrome, neurodevelopmental disability, seizures, cardiomyopathy, liver disease, fatigue, and lactic acidemia. While most DLD disease symptoms are attributed to dysfunction of the pyruvate dehydrogenase complex, the effects of other α-keto acid dehydrogenase deficiencies remain unclear. Current therapies for DLD deficiency are ineffective, with no vertebrate animal model available for preclinical study. We created a viable *Danio rerio* (zebrafish) KO model of DLD deficiency, *dldh^cri3^*. Detailed phenotypic characterization revealed shortened larval survival, uninflated swim bladder, hepatomegaly and fatty liver, and reduced swim activity. These animals displayed increased pyruvate and lactate levels, with severe disruption of branched-chain amino acid catabolism manifest as increased valine, leucine, isoleucine, α-ketoisovalerate, and α-ketoglutarate levels. Evaluation of mitochondrial ultrastructure revealed gross enlargement, severe cristae disruption, and reduction in matrix electron density in liver, intestines, and muscle. Therapeutic modeling of candidate therapies demonstrated that probucol or thiamine improved larval swim activity. Overall, this vertebrate model demonstrated characteristic phenotypic and metabolic alterations of DLD disease, offering a robust platform to screen and characterize candidate therapies.

## Abstract 2

A viable vertebrate model of DLD deficiency displays characteristic phenotypic and metabolic alterations of human *DLD^-/-^* disease, allowing screening and characterization of candidate therapies.

## Graphical abstract

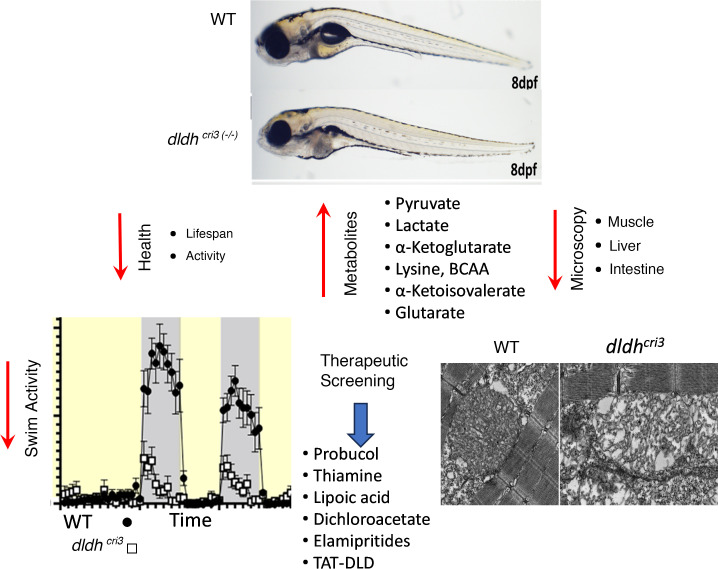

## Introduction

Dihydrolipoamide dehydrogenase (DLD or Dldh in zebrafish) is a key enzyme in mitochondrial metabolism that functions as the DLD subunit of the glycine cleavage system (GCS) (1, 2) and the E_3_ subunit of 4 α-keto acid dehydrogenase complexes in the mitochondrial matrix: pyruvate dehydrogenase (PDHc), α-ketoglutarate dehydrogenase (AKGDHc), α-ketoadipate dehydrogenase (AKADHc), and branched-chain α-keto (or 2-oxo) acid dehydrogenase (BCKADHc), which are involved in glucose metabolism, the TCA cycle, and amino acid catabolism. Autosomal recessive *DLD* mutations result in a rare primary mitochondrial disease (PMD) that manifests as Leigh syndrome, neurodevelopmental deficits, movement disorders, seizures, ataxia, cardiomyopathy, liver disease, failure to thrive, and lactic acidemia (3, 4). Although generally attributed to PDHc deficiency, the biochemical basis for the complex disease phenotype remains largely unknown. As a result, current therapies are nonspecific and generally ineffective.

Most studies of DLD deficiency are conducted on cultured cells (5), which lack the physiological complexity that differentially accrues in an organism’s organs and tissues. While we previously established an invertebrate animal model of DLD deficiency in *C*. *elegans* (6) and demonstrated its potential to be used for preclinical drug screening, development of vertebrate model organisms to study DLD deficiency will provide additional insight into both disease pathophysiology and organ-specific therapeutic development. However, vertebrate models are lacking; homozygous deletion of *Dld* in mice is embryonic lethal (7), while heterozygous *Dld^+/–^* mice do not demonstrate marked pathology (7, 8), consistent with DLD deficiency being an autosomal recessive disease. As *Danio rerio* (zebrafish) have the same major organs and tissues as humans, with the added advantage that the transparency of the larvae enables the organs to be easily observed with a dissecting microscope, the study of this model organism has intrinsic value for studying multisystemic disorders and is now widely utilized to pursue mechanistic preclinical disease studies and therapeutic modeling (9, 10). Therefore, we report on the characterization of a model of primary DLD deficiency utilizing a *dldh^cri3^* heterozygous-null mutation strain (*dldh^+/–^*) of zebrafish generated using CRISPR/Cas9 technology that is viable to adulthood and, upon mating, produces viable homozygous larvae (*dldh^–/–^*).

Detailed phenotypic characterization of *dldh^–/–^* zebrafish was performed for survival, growth, development, organ morphology, and neuromuscular swimming activity. Immunoblot analysis confirmed that *dldh^–/–^* zebrafish had substantially reduced DLD protein expression and E_3_ enzyme activity. Extensive evaluation of intermediary metabolism was performed relative to WT siblings to quantify levels in *dldh^–/–^* animals of lactate, pyruvate, and upstream metabolites of DLD-dependent α-keto acid dehydrogenase complexes as well as TCA metabolites. Organ-level morphology and mitochondrial ultrastructure was studied in the *dldh^–/–^* animal liver, intestine, and fast-twitch skeletal tail muscle. In addition, therapeutic modeling of multiple candidate therapies was performed in the *dldh^–/–^* zebrafish larval model, including a range of cofactors, small molecules used empirically or in clinical development, and enzyme-replacement therapies.

## Results

### Generation of the dldh^cri3^ zebrafish model.

**Figure 1:**
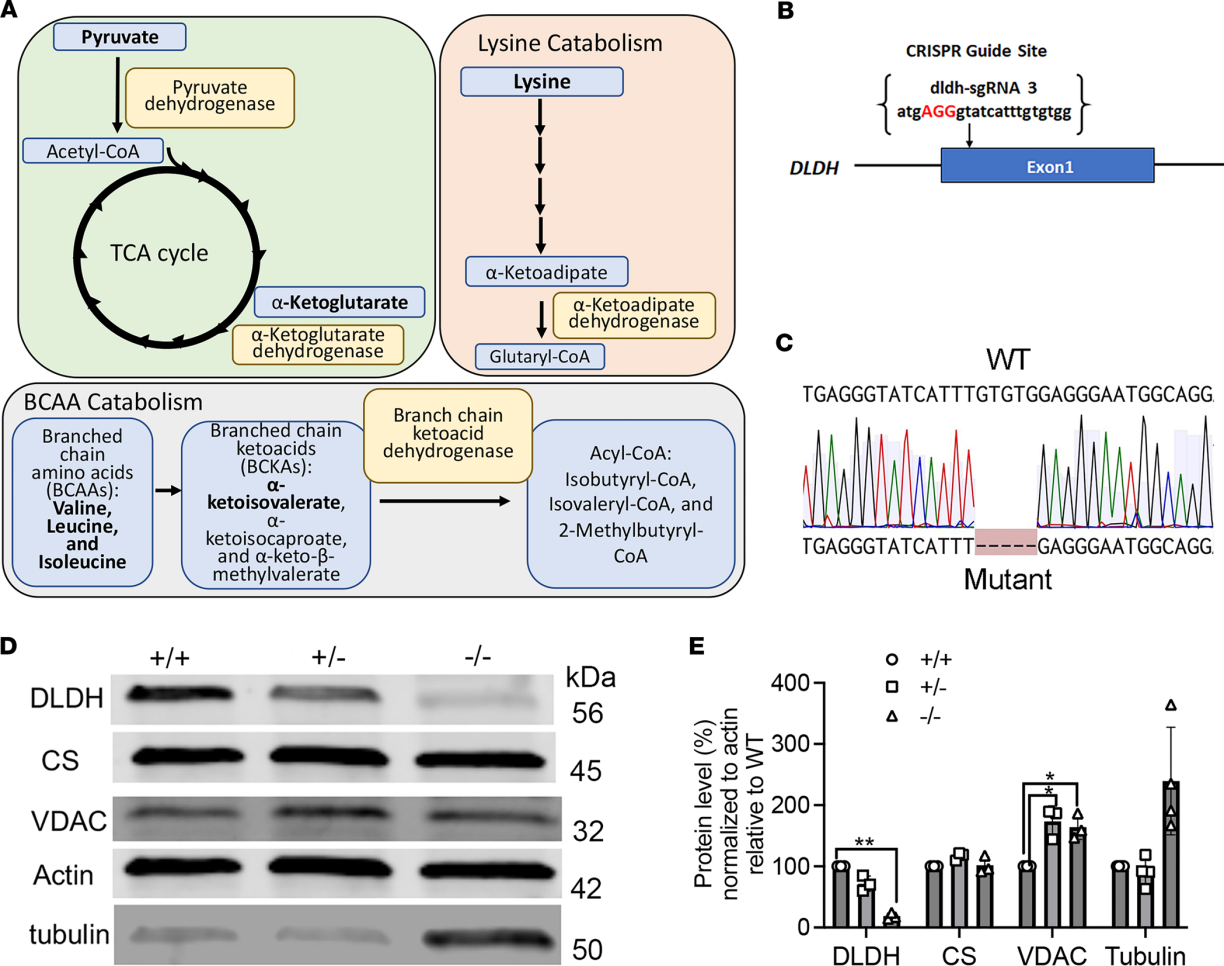
DLD role in metabolic pathways, generation of the *dldh^–/–^* zebrafish model, and confirmation of Dldh protein deficiency in *dldh^–/–^* zebrafish larvae. (**A**) Scheme representing enzyme complexes incorporating a functional DLD (E_3_) subunit: PDHc is the gateway enzyme to the TCA cycle, and AKADHc and BCKADHc are essential enzymes in lysine and BCAA catabolism, respectively. (**B**) CRISPR/Cas9 construction of *dldh^–/–^* zebrafish. Schematic showing where sgRNA targets exon 1 after start codon (ATG); the sgRNA was injected 1 hpf. (**C**) Sanger confirmation of 5 bp deletion in *dldh^cri3^* zebrafish. (**D**) Immunoblot of Dldh protein level in 7 dpf WT, *dldh^+/–^*, and *dldh^–/–^* larvae. (**E**) ImageJ quantification of Dldh, CS, VDAC, and tubulin protein levels in WT vs. 7 dpf *dldh^–/–^* larvae, showing significantly decreased Dldh expression in *dldh^–/–^* larvae and increased VDAC levels in *dldh^+/–^* and *dldh^–/–^; n* = 3; *P < 0.05 and **P < 0.01, unpaired Student’s *t* test with the Bonferroni correction. Data are shown as mean ± SD.

DLD is the enzyme responsible for the NAD^+^-dependent oxidation of dihydrolipoamide to lipoamide, a covalently bound cofactor in 4 mitochondrial enzyme complexes. Figure 1A shows the enzymes that require DLD: PDHc, AKGDH, BCKADHc, and AKADHc. The zebrafish harbor a single *DLD* gene orthologue *dldh* encoding Dldh, which has 91% aa sequence identity to human DLD (Supplemental Figure 1; supplemental material available online with this article; https://doi.org/10.1172/jci.insight.178973DS1).

We generated a stable KO zebrafish model of DLD deficiency, *dldh^cri3^*, by using CRISPR/Cas9 technology (11) in AB WT zebrafish (AB strain; https://zfin.org/action/genotype/view/ZDB-GENO-960809-7) by targeting a *dldh* sequence (Figure 1B). The heterozygous *dldh^cri3^* line was confirmed by Sanger sequencing (Figure 1C), which identified the presence of a 5 bp deletion causing the truncation of the protein at aa 371 in homozygous larvae (c.1180_1184delGTGTG, p.C371X). We confirmed Dldh protein deficiency in 7 dpf *dldh^–/–^* zebrafish larvae by immunoblot analysis (Figure 1D and Supplemental Figure 2). Indeed, Dldh protein level was reduced by > 90% (*P* < 0.001) in 7 dpf *dldh^–/–^* zebrafish larvae as compared with the relative WT control larvae (Figure 1, D and E). Furthermore, we analyzed expression levels of mitochondrial proteins citrate synthase (CS) and voltage-dependent anion channel (VDAC) as well as tubulin, which are implicated in regulating the architecture and dynamics of the mitochondrial network (12, 13). While CS protein levels were unchanged among 7 dpf *dldh^–/–^, dldh^+/–^*, and *dldh^+/+^* larvae, VDAC levels were higher in both *dldh^+/–^* and *dldh^–/–^* larvae as compared with control; this may indicate increased mitochondrial mass. In addition, tubulin protein levels trended higher in *dldh^–/–^* zebrafish larvae relative to WT animals, and this may be indicative of adaptive changes in the microtubule network. Since β-actin levels did not change in the mutant lines relative to controls, all protein expression levels were normalized to β-actin as a loading control. Overall, these data confirm successful creation of a viable genetic vertebrate model of DLD deficiency, *dldh^cri3^*, which can be stably maintained in the heterozygous state and incrossed to obtain homozygous mutant animals for study.

### Biochemical characterization of Dldh-deficient zebrafish larvae.

**Figure 2:**
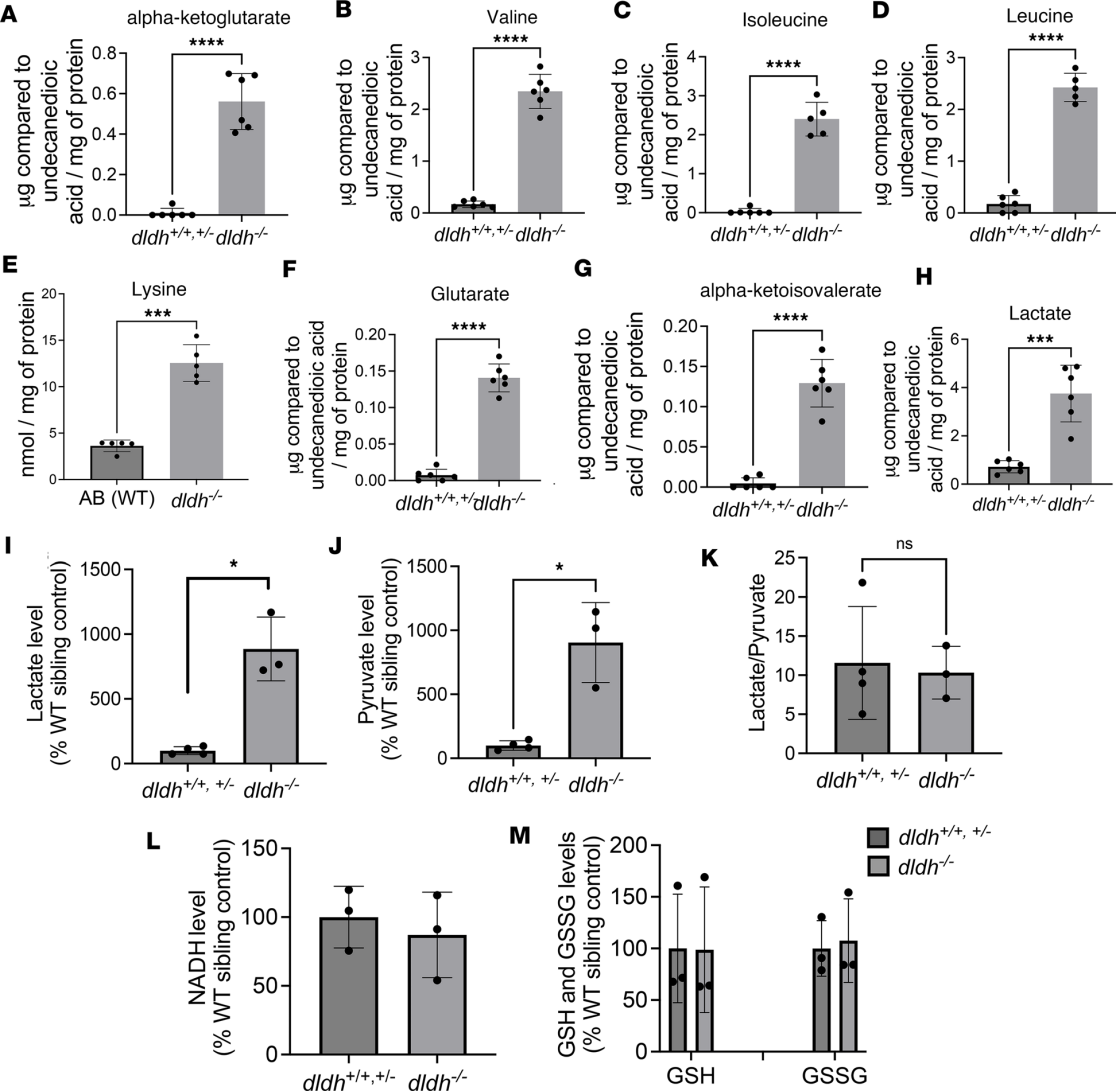
DLD-dependent enzyme metabolite analysis and mitochondrial function in *dldh^–/–^* zebrafish larvae. (**A**–**H**) Biochemical analysis by GC/MS of DLD-dependent enzyme metabolite and aa, relative to undecanoic acid standard and normalized to protein α-ketoglutarate, valine, isoleucine, leucine, lysine, glutarate, α-ketoisovalerate, and lactate in 7 dpf WT (*dldh^+/+^* and *dldh^+/–^*)and *dldh^–/–^* zebrafish larvae. *n* = 5 biological replicates/condition. ***P < 0.001; ****P < 0.0001; Welch’s unpaired *t* test. Data are shown as mean ± SD. (**I**–**K**) Lactate and pyruvate, analyzed using lactate and pyruvate oxidase-based colorimetric assays, in 7 dpf *dldh^–/–^* zebrafish larvae. Data were normalized to WT sibling controls. *P < 0.05. Student’s *t* test. (**L** and **M**) Analysis of NADH and GSH and GSSG levels (percentage of control) showed no differences between *dldh^–/–^* and WT larvae at 7 dpf. All colorimetric analyses were conducted on 4 WT and 3 *dldh^–/–^* larval biological replicate samples. Data are shown as mean ± SD.

**Figure 3:**
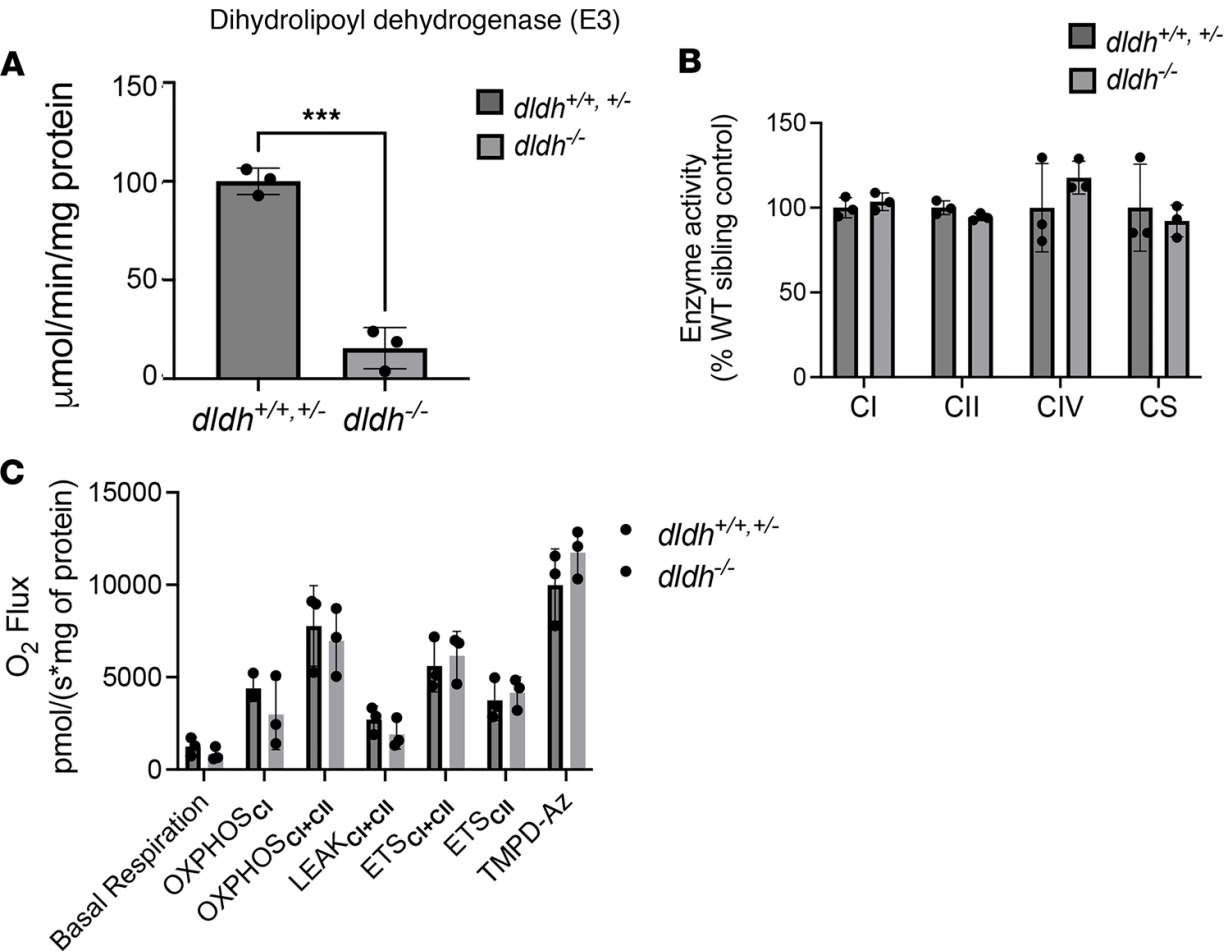
Unchanged RC enzyme activity and mitochondrial respiration capacity in *dldh^–/–^* zebrafish larvae. (**A**) Measurement of the DLD (E_3_) activity in *dldh^–/–^* zebrafish showed significantly decreased activity as compared with WT; *n* = 3; ***P < 0.001 by Student’s *t* test. (**B**) Enzyme activities of complex I (CI), CII, and CIV and citrate synthase (CS); *n* = 3. Data are shown as mean ± SD. No significant differences between 7 dpf *dldh^/^* larvae and WT by Student’s *t* test. (**C**) Oxygen flux measurements obtained by high-resolution polarography with an Oxygraph-2k (Oroboros) on 5 dpf zebrafish homogenate. Basal respiration is the measurement before addition of substrates. OXPHOS_CI_ is the capacity of CI taken after the addition of glutamate. OXPHOS_CI+CII_ is the capacity of both CI+CII taken after the addition of succinate. LEAK_CI+CII_ is the nonphosphorylating electron transfer across the mitochondrial inner membrane. ETS_CII_ is in the presence of succinate and rotenone. TMPD-Az is the ascorbate driven complex IV activity. A typical time course and experimental details are presented in Supplemental Figure 3C. None of the differences reached statistical significance by Welch’s *t* test. Data are shown as mean ± SD (*n* = 3).

Biochemical analyses were performed to confirm the expected increase in precursor metabolites for DLD-dependent enzymes. As anticipated, loss of function for these reactions significantly increased their respective enzymatic substrates as measured by gas chromatography/mass spectrometry (GC/MS) analysis in zebrafish larvae (Figure 2). BCKADHc deficiency was confirmed by identifying increased valine, isoleucine, leucine, and α-ketoisovalerate levels; AKGDHc deficiency in *dldh^–/–^* larvae was confirmed by identification of elevated levels of the TCA intermediate, α-ketoglutarate; and AKADHc deficiency was confirmed using HPLC by identifying a more than 3-fold elevation of lysine due to the block in lysine catabolism. We detected more than 4-fold elevated lactate levels using both GC/MS (Figure 2, A–H) and colorimetric assays (Figure 2I), with a similar degree of increased pyruvate levels (Figure 2J), leading to an unchanged lactate/pyruvate ratio as compared with controls (Figure 2K), consistent with what is classically associated with PDHc deficiency (14). No significant differences were seen in NADH, glutathione (GSH), or glutathione disulfide (GSSG) levels in the *dldh^–/–^* larvae as compared with controls (Figure 2, L and M). ATP and NAD^+^ levels were also unchanged (Supplemental Figure 3, A and B). Thus, biochemical analyte analysis confirmed significant metabolic disruption involving all E_3_-containing mitochondrial enzyme complexes in Dldh-deficient zebrafish larvae. Overall, E_3_ enzyme activity was significantly reduced by an average of 85% relative to WT controls (*P* < 0.001; Figure 3A).

In contrast to DLD substrates, mitochondrial RC enzymatic activity measured spectrophotometrically was not significantly affected in *dldh^–/–^* larvae at 7 dpf as compared with control (Figure 3B), in agreement with independent data obtained from polarographic analyses by high-resolution oxygen flux measurements (*J*_O2_) (Figure 3C and Supplemental Figure 3C). Indeed, *dldh^–/–^* zebrafish showed no significant changes in *J*_O2_ at basal respiration. Following addition of the designated substrates, uncouplers, or inhibitors, oxidative phosphorylation (OXPHOS) complex I substrate–dependent coupled rate (OXPHOS_CI_ rate), complexes I+II substrate–dependent coupled rate (OXPHOS_CI+CII_ rate), RC complex I through complex IV activity uncoupled from ATP synthesis (ETS_CI+CII_ rate), and isolated complex IV activity (TMPD-Az rate) in 5 dpf zebrafish larvae were not significantly different between WT and dldh^–/–^ zebrafish (Figure 3C). While glutamate was added as a complex I substrate, the requirement for an intact TCA cycle may be obviated by the presence of excess pyruvate and malate. These measurements indicate that RC capacity is not affected by the *dldh^–/–^* mutation with significantly decreased Dldh expression and E_3_ activity. Furthermore, these data imply that downstream pathways affected by loss of E_3_-containing mitochondrial enzyme complexes do not have a major effect on RC complex enzymatic activities or integrated OXPHOS capacity.

### Impaired survival, gross anatomy, and swim activity in dldh^–/–^ larvae.

**Figure 4:**
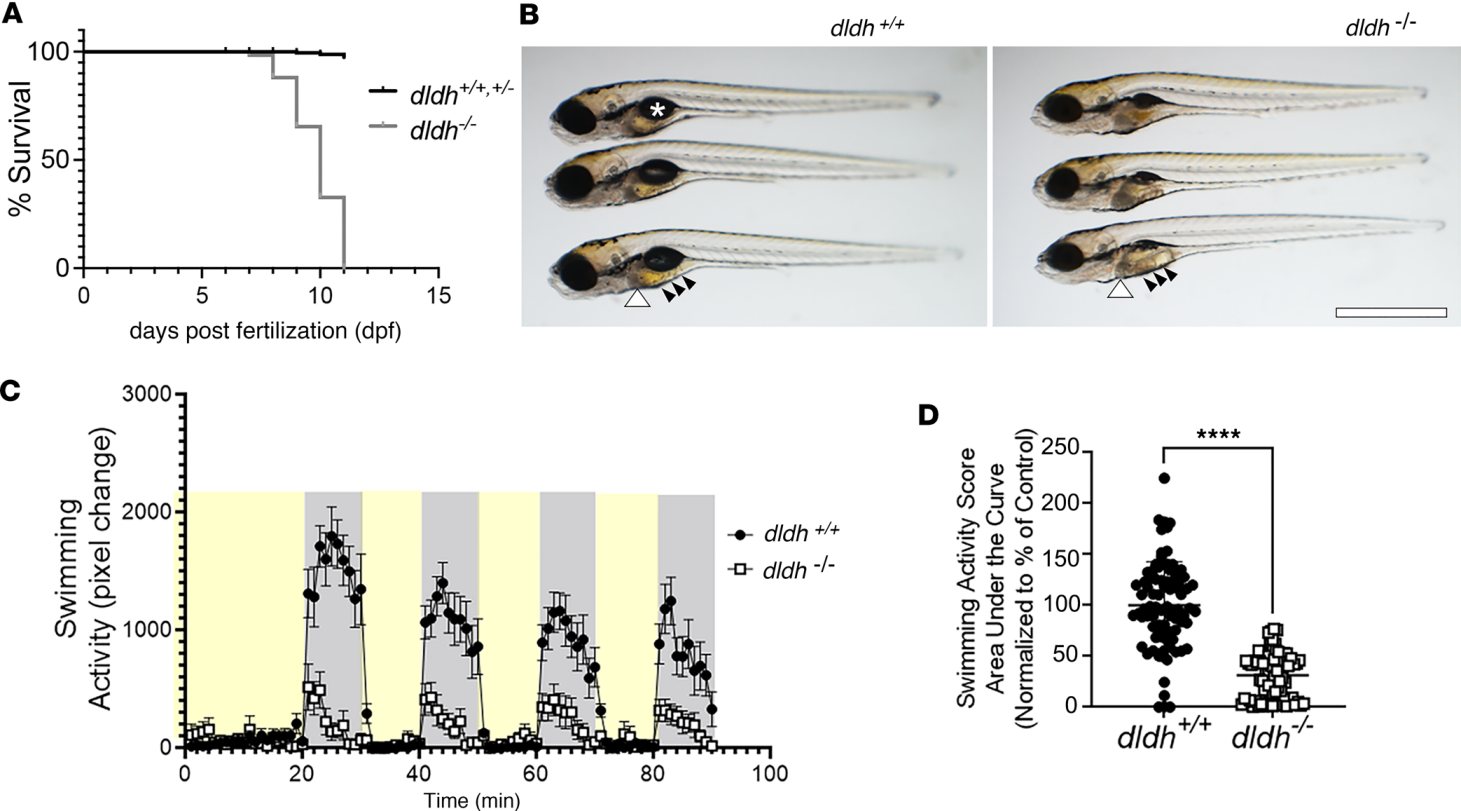
Decreased survival, abnormal gross morphology, and reduced swimming activity in *dldh^–/–^* zebrafish larvae. (**A**) *dldh^–/–^* larvae had reduced survival relative to WT larvae, with mortality starting at 8 dpf; *n* = 300 each strain, across 3 biological replicates; log-rank (Mantel-Cox) test: *P* < 0.0001; median survival of *dldh*^–/–^: 10 dpf. Death was defined by absent heartbeat. (**B**) Morphological analysis at 7 dpf showed *dldh^–/–^* zebrafish larvae had enlarged liver (white arrowhead), darker intestine (black arrows) with universal finding of uninflated swim bladder (asterisk in WT), relative to WT larvae. Scale bar: 0.5 mm. (**C**) Larval swimming activity (ZebraBox platform and ZebraLab software) tracing showed reduced *dldh^–/–^* mutant swimming (open circles) in dark periods (gray) at 7 dpf. Larvae were exposed to 3 consecutive 10-minute 100% light (yellow) and 10-minute dark cycles following a light/dark acclimation period. Each point shows average (mean + SEM) activity of 84 larvae/strain. (**D**) *dldh^–/–^* swimming activity at 7 dpf during dark cycles analyzed by AUC, with group comparison. ****P < 0.0001 by unpaired Student’s *t* test. Data are shown as mean ± SEM. *n* = 84 larvae across > 3 biological replicate experiments per strain.

*dldh^–/–^* zebrafish larvae showed decreased survival relative to WT animals, with survival rate decreasing starting at 8 dpf, a 50% reduction in lifespan at 9 dpf, and a precipitous fall to 100% mortality registered at 13 dpf (Figure 4A). *dldh^–/–^* larvae had an average lifespan of 11 dpf, which is significantly reduced relative to WT zebrafish that survive beyond 2 years (*P* < 0.0001).

The swim bladder is a gas-filled organ essential for survival and swimming activity in most teleost species (15) and in zebrafish is generally fully developed at 5 dpf. We universally observed deflated swim bladders in *dldh^–/–^* larvae at 5 dpf, which did not develop in later stages, as compared with 5 dpf WT larvae that exhibited completely inflated swim bladders (Figure 4B). Starting from 6 dpf, *dldh^–/–^* larvae also exhibited enlarged and darker livers and intestines (Figure 4B), indicative of a pathological phenotype. Larval swimming analysis using the ZebraBox system showed that both control and *dldh^–/–^* larvae responded to a light/dark transition (increased activity in darkness; Figure 4C). However, 7 dpf *dldh^–/–^* larval swimming activity was 69% decreased (*P* < 0.0001) during dark cycles relative to controls (Figure 4D). Overall, these data confirm that the *dldh^–/–^* zebrafish derived from the *dldh^cri3^* line demonstrated severe physiologic abnormalities in the larval period, including reduced lifespan with mean survival of 9 dpf, gross morphologic changes such as deflated swim bladders seen by 5 dpf with enlarged and darkened livers and intestines by 6 dpf, and reduced swimming activity at 7 dpf.

### Liver pathology in dldh^–/–^ zebrafish larvae.

**Figure 5:**
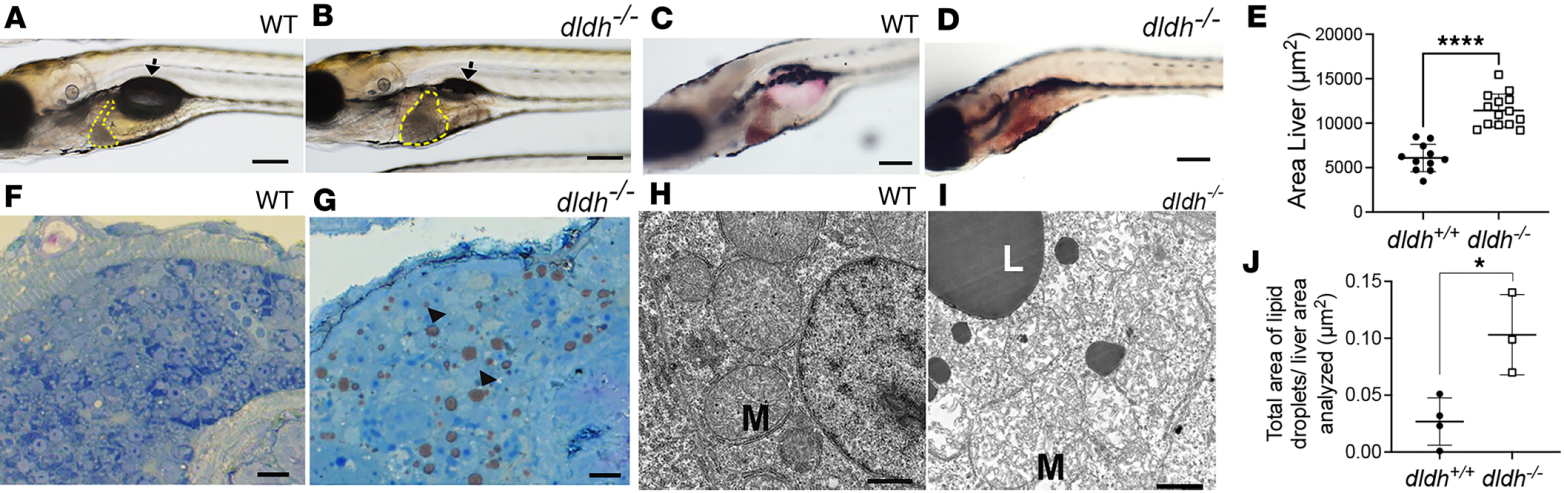
Liver pathology in *dldh^–/–^* zebrafish larvae. (**A** and **B**) WT and *dldh^–/–^* morphology at 7 dpf showed mutants had increased liver size (yellow line) and deflated swim bladder (arrow). Scale bar: 100 μm (×2.5 magnification of the middle fish in Figure 4B). (**C** and **D**) Oil Red O staining of 8 dpf WT and *dldh^–/–^* liver showed increased lipid accumulation in mutant larvae. Scale bar: 100 μm. (**E**) Liver area (μm^2^) was increased in 14 *dldh^–/–^* relative to 11 WT larvae by ImageJ analysis across 3 replicate experiments. ****P < 0.0001 by unpaired Student’s *t* test. (**F** and **G**) Thin sections of WT and *dldh^–/–^* liver stained with toluidine blue showed mutant larvae had increased frequency of lipid droplets (arrowheads). Scale bars: 10 µm. (**H** and **I**) Hepatocyte ultrastructure in 7 dpf WT and *dldh^–/–^* showed mutant larvae had mitochondrial damage (M) and large lipid droplets (L). Scale bars: 1 µm. (**J**) Ultrastructural analysis showed increased fractional lipid droplet to liver area analyzed in 4 WT and 3 *dldh^–/–^* larvae at 7 dpf. Areas determined using ImageJ. *P < 0.05 by unpaired Student’s *t* test.

*dldh^–/–^* zebrafish larvae showed hepatomegaly, with significantly increased liver size at 7 dpf relative to WT larvae (*P* < 0.0001) (Figure 5, A, B, and E). Oil Red O staining of *dldh^–/–^* larvae at 8 dpf showed increased red staining in the liver as compared with WT larvae, indicating lipid accumulation (Figure 5, C and D). Indeed, *dldh^–/–^* larvae displayed an increased rate of having large lipid droplets in 7 dpf hepatocytes that were absent in WT liver. An increased lipid droplet rate was shown by histological examination of thin sections stained with toluidine blue and ultrathin transmission electron microscopy (TEM) sections (Figure 5, F–I). The total hepatocyte area occupied by lipid droplets was significantly increased in 7 dpf mutant larvae relative to WT (*P* < 0.05) (Figure 5J). TEM analysis of the mutant larvae at 7 dpf also showed mitochondrial damage, a decreased number of cristae with abnormal structure, and decreased matrix electron density (16, 17) resulting from reduced intercristae electron density (Figure 5, H and I). Collectively, these data demonstrate that *dldh^–/–^* zebrafish larvae have hepatomegaly and fatty liver, with increased large lipid droplets and abnormal mitochondrial ultrastructure.

### Quantitative analyses of mitochondrial ultrastructure abnormalities in 7 dldh^–/–^ larvae in 3 high-energy demand tissues.

Given the gross appearance of abnormal mitochondria ultrastructure seen in *dldh^–/–^* larvae livers, quantitative mitochondrial ultrastructure analyses were performed in high-energy–demand tissues (liver, intestine, and skeletal muscle tail fibers) in *dldh^–/–^* larvae, at 7 dpf. We classified the mitochondrial ultrastructure phenotypes observed as “normal”, “damaged”, and “degenerated”. “Normal” indicates mitochondria exhibiting cristae, matrix electron density, and shape integrity. “Damaged” and “degenerated” classifications both indicate abnormal mitochondrial ultrastructure with loss of cristae integrity, matrix electron density, and/or shape integrity; however, degenerated mitochondria completely lost the integrity of the cristae, external membrane, and shape. Breaks in the mitochondrial-delimiting membranes were also encountered in degenerated mitochondria.

### Hepatocyte mitochondrial ultrastructure.

**Figure 6:**
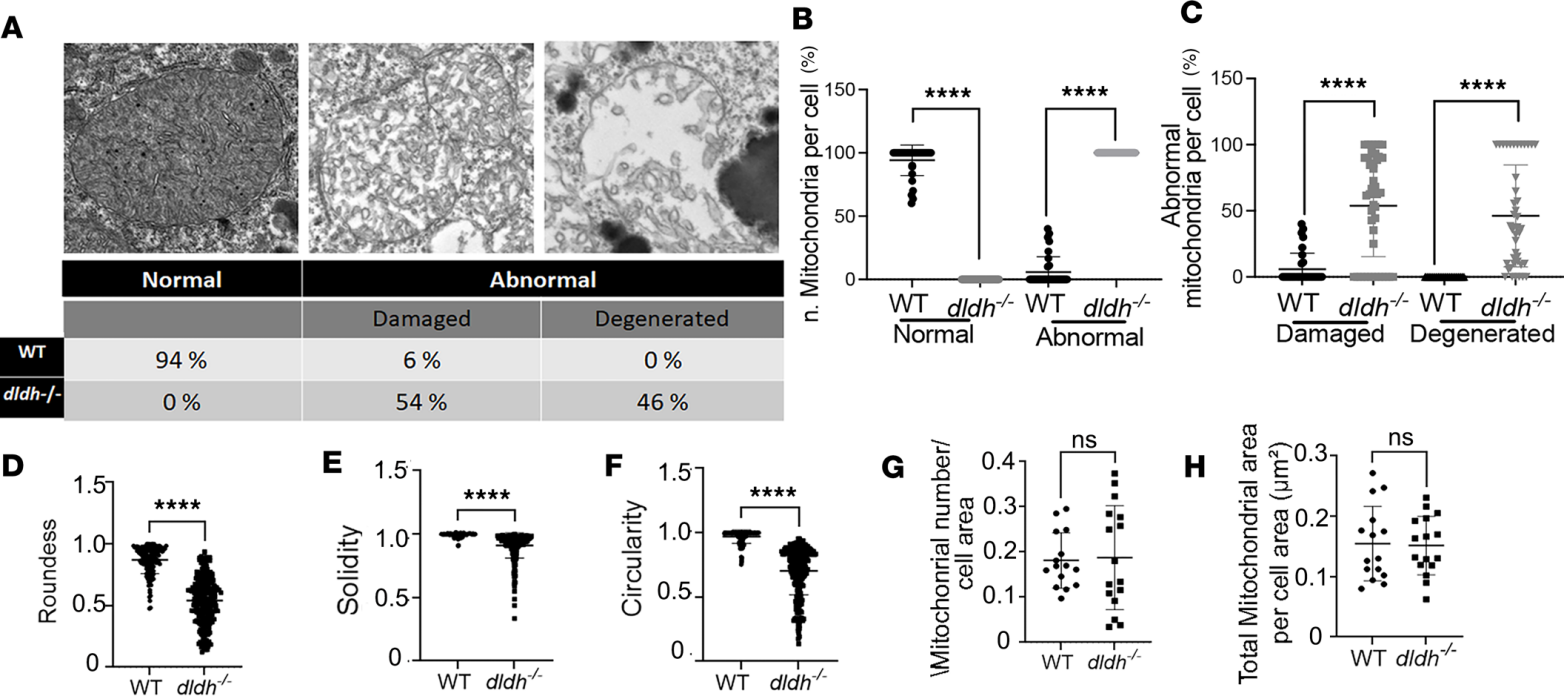
Mitochondrial ultrastructure changes in 7 dpf *dldh^–/–^* liver mitochondria. (**A**) “Normal” and “abnormal” (damaged and degenerated) ultrastructure representative images above summary table for analysis of 3 WT and 3 *dldh^–/–^* zebrafish from 34 (WT) and 43 (*dldh^–/–^*) cells and 209 (WT) and 635 (*dldh^–/–^*) mitochondria analyzed (the “damaged” mitochondrion is present in Figure 5I image). (**B**) Scatter plot showing percent of normal and abnormal mitochondria per cell, as described in **A**. Data are shown as mean ± SD. (**C**) Percent of abnormal mitochondria classified as damaged or degenerated, as described in **A**. (**D**–**F**) Morphological features analyzed: roundness, solidity, and circularity quantifying mitochondrial shape integrity in WT and *dldh^–/–^* larvae. (**G**) Mitochondria number relative to cell area (μm^–2^). Points indicate mitochondria number per cell. (**H**) Total mitochondrial area relative to cell area. (**D**–**H**) Mitochondria, *n* = 148 (WT) and 245 (*dldh^–/–^*); cells, *n* = 15 (WT) and 16 (*dldh^–/–^*). Data are shown as mean ± SD. All statistical analyses by unpaired Student’s *t* test. ****P < 0.0001.

Mitochondrial ultrastructure was particularly damaged in *dldh^–/–^* hepatocytes (Figure 6). Overall, compared with 94% of mitochondria being classified as “normal” in 7 dpf WT hepatocytes, 100% of mitochondria were “abnormal” in 7 dpf *dldh^–/–^* hepatocytes; statistically significant differences (*P* < 0.0001) are summarized in Figure 6, A–C. Analyses of several morphological features, such as roundness, circularity, and solidity (compact shape) — which are indicative of mitochondrial shape integrity (18) — showed significant differences among WT and *dldh^–/–^* hepatocytes at 7 dpf (*P* < 0.0001) (Figure 6, D–F). Specifically, *dldh^–/–^* larvae showed markedly disrupted mitochondrial integrity relative to WT larvae, with loss of the typical roundness and circularity and less solidity. These features provide important information about both mitochondrial damage and dynamics (18). While the liver mitochondria of *dldh^–/–^* larvae were notably damaged, the number of mitochondria per liver cell area was not significantly changed when compared with WT (Figure 6G). Additionally, mitochondrial mass per cross-sectional area was not different between *dldh^–/–^* and WT larvae at 7 dpf (Figure 6H).

### Intestinal cells’ mitochondrial ultrastructure.

**Figure 7:**
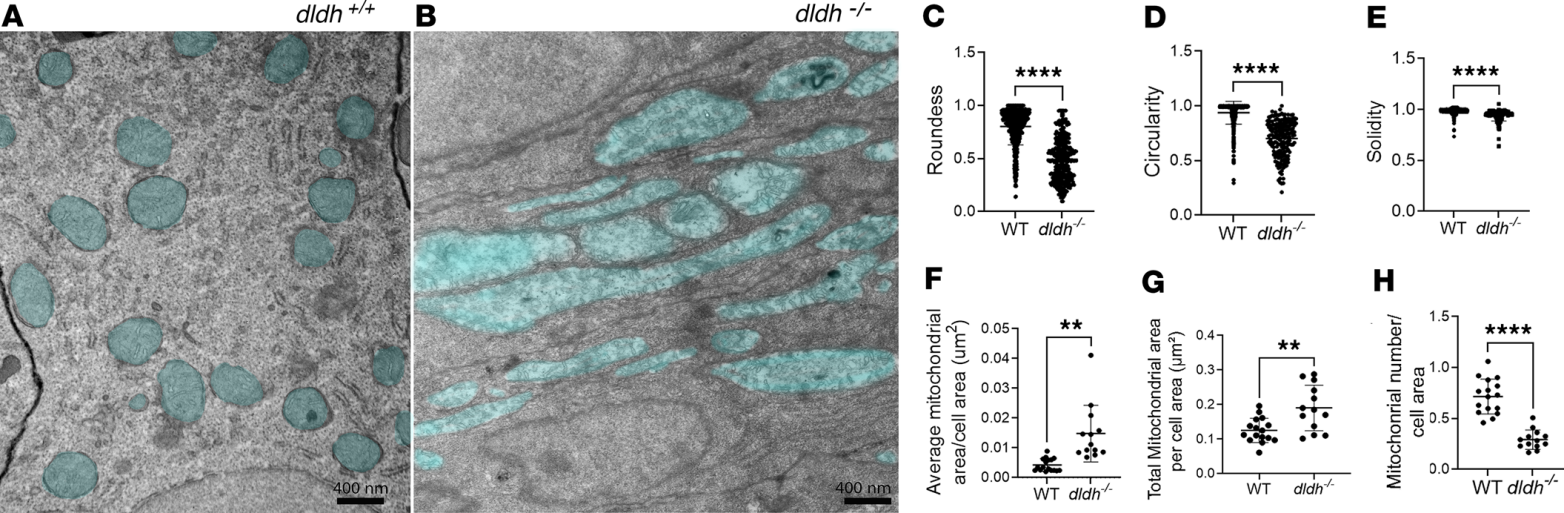
Intestinal mitochondria in 7 dpf *dldh^–/–^* larvae showed depletion of mitochondria with matrix chamber swelling and elongation. (**A** and **B**) Mitochondria ultrastructure (light blue) was nicely rounded in WT intestinal cells with well-organized rough reticulum (**A**), while intestinal cells of 7 dpf *dldh^–/–^* zebrafish larvae showed mitochondria (light blue) swelling, damage, and elongation (**B**). (**C**–**E**) Mitochondria ultrastructural morphological features quantified in intestinal cells of WT and *dldh^–/–^* larvae mutant larvae. Mitochondria, *n* = 504 (WT) and 190 (*dldh^–/–^*) in 16 cells (WT) and 13 cells (*dldh^–/–^*) from 3 WT and 3 *dldh^–/–^* larvae. (**F**–**H**) Average mitochondrial area, total mitochondrial area, and mitochondria number relative to cross-sectional area . Analysis confirmed increased mitochondrial area and decreased number of mitochondria in *dldh^–/–^* larvae relative to WT. All characteristics determined with ImageJ; statistical analyses by Student’s *t* test, and each point represents data from 1 cell. ****P < 0.0001; **P < 0.01. Data are shown as mean ± SD.

Mitochondrial ultrastructure abnormalities were evident in intestinal cells in *dldh^–/–^* 7 dpf larvae. Zebrafish intestinal cells in WT animals typically have rounded mitochondria and a well-organized rough reticulum. While this mitochondrial structure was observed in 7 dpf WT larvae (Figure 7A), intestinal cell mitochondria in 7 dpf *dldh^–/–^* were swollen, elongated, and damaged and had a disorganized rough reticulum (Figure 7B). This phenotypic appearance of intestinal cell mitochondrial ultrastructure closely aligned with quantitative measurements that showed significantly decreased roundness, circularity, and solidity, collectively indicating a loss of shape integrity (Figure 7, C–E). Interestingly, the average mitochondrial area (Figure 7F) and total mitochondrial area (Figure 7G) per cross-sectional area were both significantly increased by more than 50% relative to WT larvae (*P* < 0.01), while the number of mitochondria per cross-sectional cell area was significantly decreased in *dldh^–/–^* larvae (*P* < 0.01) (Figure 7H), suggestive of mitochondrial depletion. These findings suggest that increased fusion of mitochondria and/or loss of mitochondria through mitophagy occurs in *dldh^–/–^* larvae intestinal cells, as may plausibly result from water influx that would result in mitochondrial swelling (19).

### Fast-twitch skeletal muscle fiber mitochondrial ultrastructure.

**Figure 8:**
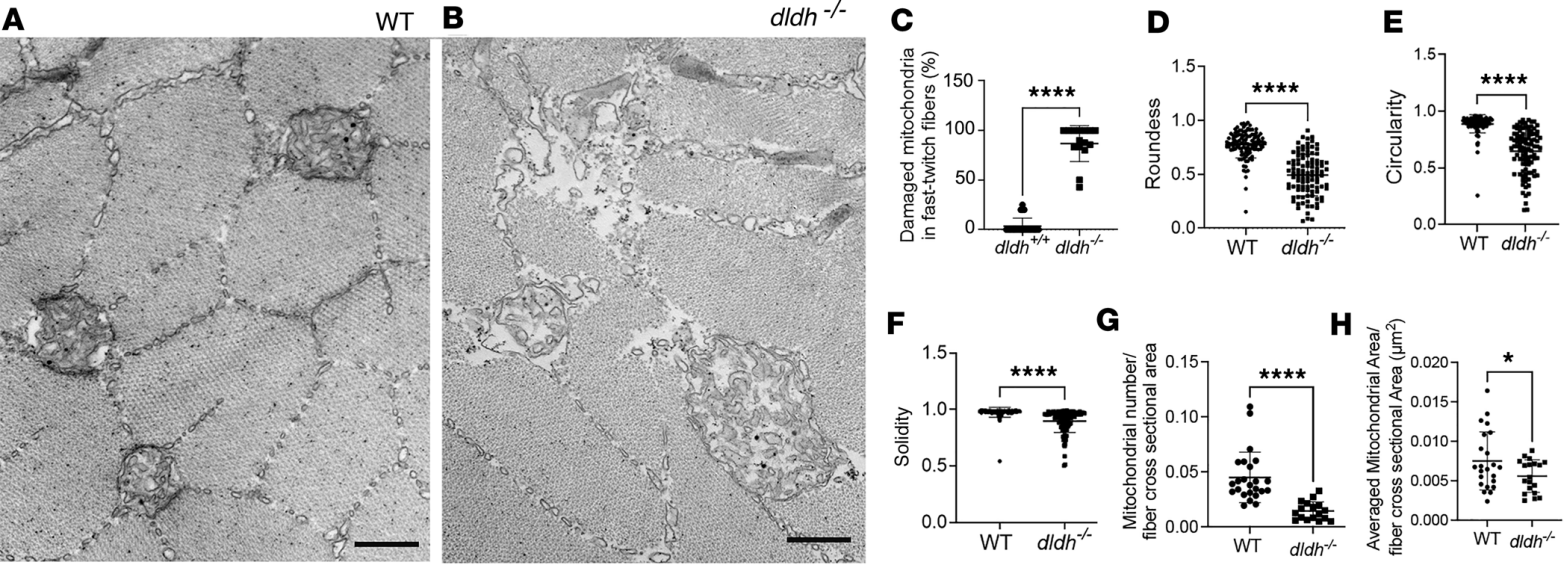
Mitochondria ultrastructural changes in 7 dpf *dldh^–/–^* larvae fast-twitch skeletal muscle fibers. (**A** and **B**) Mitochondrial ultrastructure was grossly disrupted in 7 dpf *dldh^–/–^* fibers, as compared with WT. Scale bars: 500 nm. (**C**) Percentage of damaged mitochondria in WT and *dldh^–/–^* fibers. (**D**–**F**) Mitochondrial ultrastructure morphological features of roundness, circularity, and solidity were quantitatively analyzed using ImageJ. (**G** and **H**) Mitochondria number relative to fiber cross-sectional area (μm^–2^) and average mitochondrial area per fiber cross-sectional area were analyzed using imageJ. In total, 110 (WT) and 102 (*dldh^–/–^*) in 24 (WT) and 15 (*dldh^–/–^*) fast-twitch muscle fibers from 3 WT and 3 *dldh^–/–^* larvae were analyzed. ****P < 0.0001, *P < 0.05 by unpaired Student’s *t* test. Data are shown as mean ± SD.

Since swimming activity was decreased in *dldh^–/–^* larvae at 7 dpf relative to WT, mitochondrial ultrastructure was carefully analyzed in skeletal tail muscle. Initial TEM observations showed a greater effect on mitochondrial structure in the fast-twitch fibers in the skeletal muscle tail than in the slow-twitch fibers of *dldh^–/–^* larvae. Fast-twitch fibers undergo fast contraction, have greater reliance on anaerobic glycolysis for ATP generation (20), are involved in the quick movement response, and rapidly fatigue. Mitochondria in fast-twitch fibers are mostly localized in the intermyofibrillar space, while mitochondria in slow-twitch fibers are primarily localized in the subsarcolemmal area. In 7 dpf *dldh^–/–^* larval fast-twitch fibers, less dramatic mitochondrial ultrastructural damage was observed as compared with their liver and intestine cells. Nonetheless, fast-twitch muscle fiber mitochondrial ultrastructure was significantly abnormal relative to WT larvae, showing disarranged cristae, greater intercristae lamellae distances, reduced matrix electron density, and impaired morphological integrity (Figure 8, A and B). Quantitative analysis confirmed statistically significant differences in the fraction of damaged mitochondria; an average of 83% of mitochondria showed damage in *dldh^–/–^* larvae fast-twitch muscle fibers relative to an average of 3% of damaged mitochondria in WT larvae (*P* < 0.0001) (Figure 8C). Significant abnormalities (*P* < 0.0001) were also seen in mitochondrial ultrastructural morphology, with reduced roundness, solidity, and circularity in *dldh^–/–^* larvae fast-twitch muscle fiber mitochondria (Figure 8, D–F). Mitochondrial number normalized to cross-sectional cell area was significantly lower by 50% (*P* < 0.0001) (Figure 8G), and mitochondrial area per fiber cross-sectional area was mildly decreased (*P* < 0.01) (Figure 8H), consistent with mitochondrial depletion occurring in *dldh^–/–^* larvae fast-twitch muscle fiber mitochondria.

### Preclinical drug screening on swim activity, mitochondrial metabolism, and liver pathology.

**Figure 9:**
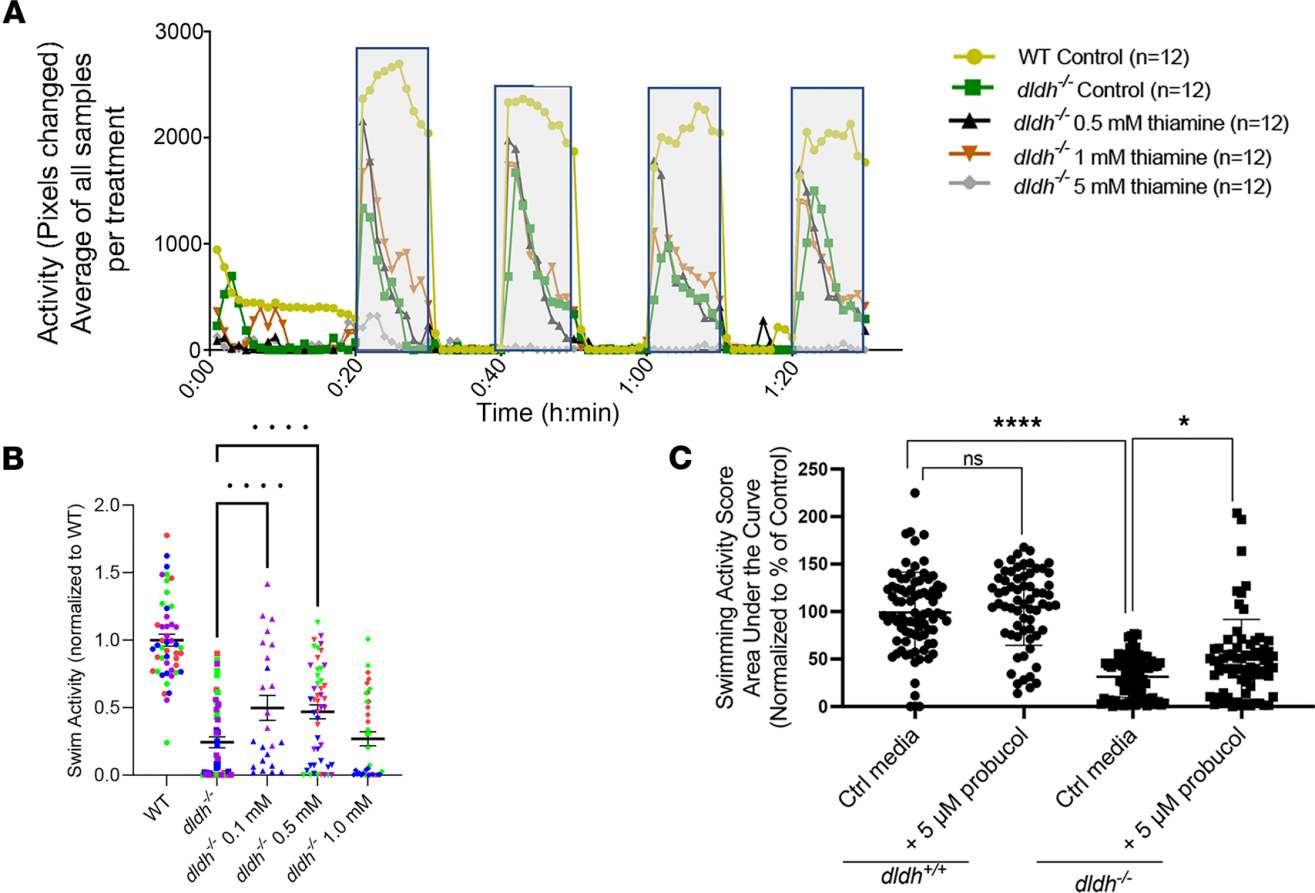
Preclinical candidate drug screening in *dldh^–/–^* zebrafish larvae. (**A**) Representative ZebraBox activity time course as in Figure 4C for WT (olive), *dldh^–/–^* (green)*,* and *dldh^–/–^* treated with 0.5, 1.0, or 5.0 mM thiamine (black, orange, gray), respectively. Notably, at 5.0 mM thiamine, there was minimal observable swim activity (*n* = 3 biological replicates). (**B**) Four replicates of the effect of 0.1, 0.5, or 1 mM thiamine treatment from 4 dpf to 6 dpf on the swimming activity of *dldh^–/–^* zebrafish larvae. Quantitation across the dark cycles was by AUC. Four biological replicates are represented by coloring the data points red, green, blue, or purple. One-way ANOVA with multiple comparisons to *dldh^–/–^* identified treatment with either 0.1 or 0.5 mM as enhancing the swimming activity of the *dldh^–/–^* by ~100%; **P < 0.01. (**C**) Similarly, probucol treatment (5 μM, 4–dpf) also increased swim activity of 7 dpf *dldh^–/–^* zebrafish larvae (*P *≤* 0.05 by unpaired Student’s *t* test with a Bonferroni correction) but not of WT; *n* = 5 biological replicates per condition, *n* = 12 zebrafish larvae for each condition per biological replicate experiment.

To assess candidate therapies’ preclinical efficacy for DLD deficiency in the *dldh^–/–^* zebrafish, we sought to determine if candidate therapies rescued their reduced swimming activity, metabolic abnormalities, and liver pathology. Table 1 summarizes the efficacy studies performed for 3 drugs empirically used in PDHc deficiency (thiamine, lipoic acid, dichloroacetate [DCA]) and 2 candidate therapies being developed for PMD (probucol, elamipretide) (21, 22), including concentrations and incubation times tested in *dldh^–/–^* larvae. Only incubation with low concentrations of thiamine (0.1 or 0.5 mM) or 5 μM probucol from 4 to 7 dpf significantly rescued *dldh^–/–^* larvae swimming activity (*P* < 0.001 and *P* < 0.05, respectively) (Figure 9, A–C). While 25 mM DCA did not rescue *dldh^–/–^* zebrafish larval swimming activity, it did significantly reduce their increased tissue pyruvate level and pyruvate/lactate ratio without rescuing their increased tissue lactate level (Table 1 and Supplemental Figure 4), suggesting that the mutants’ reduced swim activity was not solely attributable to PDHc dysfunction. Surprisingly, higher thiamine concentration (5 mM) appeared toxic in this model, as it eliminated all swimming activity in *dldh^–/–^* larvae (Figure 9A). Additionally, probucol did not rescue increased lactate and pyruvate levels nor the range of increased DLD-dependent enzyme metabolite substrates of *dldh^–/–^* zebrafish larvae (Supplemental Figure 4, A–F). No benefit was seen with either probucol or 2 other drugs (thiamine and lipoic acid) tested on hepatomegaly or lipid accumulation by Oil Red O staining in *dldh^–/–^* larvae liver (Table 1).

### TAT-DLD injection did not rescue the disease phenotype in dldh^–/–^ larvae.

Given published reports that TAT-DLD delivered DLD to PDHc within mitochondria of asymptomatic, heterozygous *Dldh^+/–^* mice (5, 8) and in cells from patients with DLD disease, we evaluated whether this therapeutic approach might rescue *dldh^–/–^* larvae. Embryos were injected at the 1-cell stage with 0.237 ng protein of either TAT-DLD–protein-1 or TAT-DLD–protein-2 (5, 8) in a volume of 900 pL, together with FITC-dextran (injection efficiency marker) or FITC-dextran and E_3_ media alone. Images confirmed successful injection, as visualized at 3 dpf (Supplemental Figure 5A). At 6 dpf, larvae were collected for immunoblot analysis. TAT-DLD–protein-1–injected *dldh^–/–^* larvae showed increased DLD protein fluorescence expression compared with mock-injected *dldh^–/–^* larvae (Supplemental Figure 5A). TAT-DLD–injected larvae did not show survival improvement as compared with the mock-injected larvae, nor did they show rescue of deflated swim bladder or gross liver pathology (not shown). Swimming activity at 7 dpf of TAT-DLD–protein-1– or TAT-DLD–protein-2–injected *dldh^–/–^* larvae was not improved by ZebraBox dark-cycle period analysis (Supplemental Figure 5B). While DLD enzymatic activity (E_3_) was again found to be substantially reduced in *dldh^–/–^* larvae as compared with WT larvae (Figure 3A), *dldh^–/–^* larvae injected with the TAT-DLD protein (0.237 ng injected in 900 pL, the maximum achievable concentration) showed only modestly improved E_3_ activity as compared with uninjected homozygous larvae at 7 dpf (Supplemental Figure 6). Thus, TAT-DLD was not found to rescue the gross phenotype of *dldh^–/–^* zebrafish.

## Discussion

DLD deficiency is a rare, autosomal recessive PMD that causes multisystem symptomatology that may variably include Leigh syndrome, neurodevelopmental disability, movement disorders, seizures, ataxia, cardiomyopathy, liver disease, failure to thrive, fatigue, and lactic acidemia (23). Unfortunately, no proven effective therapies or cure exist for this disease. To facilitate the understanding of the DLD disease mechanisms and enable preclinical modeling of candidate therapies for future clinical trials, it is critical to have viable animal models for study. A previously reported murine *Dld*-KO model with a homozygous gene deletion was embryonically lethal while the heterozygotes were asymptomatic, preventing an in-depth study of the DLD disease mechanism (7). We previously reported a viable invertebrate *C*. *elegans* RNAi-knockdown worm model of DLD deficiency, which demonstrated organismal pathology relevant to the human disease and can be used for candidate therapy and high-throughput drug screening (6). Here, we used CRISPR/Cas9 technology to successfully generate a viable zebrafish larval model of severe DLD deficiency, *dldh^cri3^*. This larval recessive mutation model offers the advantages of characterizing the gross disease phenotype and complex measures of intermediary metabolism in viable DLD-deficient animals, along with relevant measures of neuromuscular swimming activity and organ-level pathology, including both histologic and in-depth electron microscopy examination of mitochondrial structural impairment in high-energy–demand tissues.

The DLD-deficient *dldh^–/–^* larvae model was biochemically validated by demonstrating both Dldh protein level deficiency and the significant loss of E_3_ activity in the *dldh^–/–^* larvae. Clinically, DLD deficiency is often viewed as a form of PDHc deficiency, with little consideration of the contribution of pathogenic dysfunction present in the other E_3_-dependent metabolic pathways. Here, careful investigation was made into the metabolic pathways involving the multiple E_3_-dependent reactions: PDHc, AKGDHc, AKADHc, BCKADHc, and the GCS. Indeed, increased upstream metabolites of valine, isoleucine, leucine, lysine, α-ketoglutarate, and pyruvate were observed, implying dysfunction in branched-chain aa and lysine catabolism, the TCA cycle, and PDHc (24). With reduced PDHc activity, pyruvate levels were increased and the levels of metabolites participating in the TCA cycle were variably disrupted. Importantly, we also detected increased lactate level, one of the common symptoms observed in patients (23). Surprisingly, significantly increased glutarate was observed, which may suggest an alternative fate of the thiamin-5-hydroxypentanoic acid intermediate formed on the E_1_ subunit of AKADHc (25). If Dldh absence decreases the GCS activity, an increase in glycine might have been expected, as 594 mutations in the other 3 components of the GCS result in hyperglycinemia (26). Collectively, these data provide confirmation in a viable animal model that DLD deficiency causes disruption not just of PDHc activity but of the activity of the other α-keto acid dehydrogenases.

DLD deficiency is recognized as a PMD (27). Surprisingly, despite the observed dysfunction of the TCA cycle and PDHc in DLD deficiency in the *dldh^–/–^* zebrafish, basal mitochondrial respiration was comparable in the mutant relative to control larvae. Indeed, we show that DLD deficiency in the *dldh^–/–^* larvae did not affect mitochondrial RC enzyme activity, as demonstrated both by spectrophotometric analyses of CI, CII, and CIV enzyme activities and by the unchanged respiratory function on high-resolution oximetry analysis of the integrated RC OXPHOS complexes. This result corroborates our previously reported observation in the *dld-1(RNAi)* *C*. *elegans* model, where the knockdown of *dld-1* in the worms did not significantly alter RC enzyme activities (6). Collectively, these data suggest that, while DLD deficiency is a disorder of mitochondrial metabolism, DLD mutations do not directly affect RC-enzyme activities and suggest these forms of PMD would not be biochemically diagnosed by RC enzyme complex activity analysis in biopsied muscle tissue. Furthermore, to better understand DLD disease pathophysiology and therapeutic opportunities, the role of multiple altered metabolite concentrations will need to be considered.

Studying the *dldh^cri3^* zebrafish model, we were able to replicate major aspects of DLD disease patient symptomatology, including larval developmental delay, decreased neuromuscular swimming activity, and severely decreased survival in *dldh^–/–^* larvae relative to healthy WT animals (23). Similarly, developmental delay and impaired gross locomotor activity were also previously observed in *dld-1*(*RNAi*) *C*. *elegans* (6). The sharp decrease in survival of the *dldh^–/–^* larvae after 8 dpf is attributed to the failure of the swim bladder to inflate (Figure 5B), which is required for larval and adult swimming. Zebrafish are used for toxicology studies, where delayed swim bladder inflation is a common observation; these studies have identified ROS generation and subsequent inhibition of Wnt and Hedgehog signaling pathways as causative mechanisms (28). The role of DLD, and of mutant DLD, in increasing ROS has been investigated and previously implicated in the etiology of DLD disease (25, 29, 30). Future studies will examine ROS production in *dldh^–/–^* larvae and the potential interaction with Wnt and Hedgehog signaling. In addition, *dldh^–/–^* zebrafish larvae showed hepatomegaly, a common observation in DLD-deficient patients (31). Furthermore, the disruption of mitochondrial ultrastructure in hepatocytes along with accumulation of lipid droplets is suggestive of impaired fat metabolism, an unanticipated sequelae of DLD deficiency, since increased fat oxidation could provide an alternative source of acetyl-CoA to the TCA cycle and of NADH to the RC that may compensate for the loss of pyruvate oxidation (32).

Disrupted ultrastructure of mitochondria of varying severity was observed in all *dldh^–/–^* cells examined in the *dldh^cri3^* zebrafish larval model, including intestinal cells and fast-twitch skeletal muscle fibers at 7 dpf. Mitochondrial ultrastructure studies are particularly relevant to directly visualize and understand the mitochondrial damage caused by DLD deficiency. Indeed, extensive mitochondrial changes observed in *dldh^–/–^* larvae indicate the critical effect of the *dldh^–/–^* mutation on mitochondrial function. Further study will focus on investigating the effect of DLD deficiency on mitochondrial dynamics and mitophagy to better understand the broader cellular effect of DLD function and deficiency. Furthermore, this model enables improved opportunity for future preclinical drug screening validation studies by providing clear cell-based imaging outcomes. This deletion mutant results in the complete lack of DLD, so screening methods will potentially miss drugs with therapeutic effects that would enhance the stability or activity of the DLD present in the pathological variants arising from point mutations.

The critical mitochondrial ultrastructural changes observed in different high-energy–demand tissues of the *dldh^–/–^* larvae included breaks in the mitochondria-limiting membranes, particularly in grossly swollen mitochondria. The latter may be caused by influx of water and solutes into the outer chamber causing “ballooning” of cristae (19), particularly as was observed in hepatocytes and intestinal cells. Mitochondrial elongation was also encountered, especially in intestinal cells. Mitochondrial dynamics (fission, fusion, motility) regulate the organelle’s morphology and distribution, which when disrupted has been linked to a loss of metabolic function (33, 34). Mitochondrial disruption in the liver and intestine at 7 dpf is indicative of a final stage in the mitochondrial life, corroborated by the *dldh^–/–^* larvae morbidity starting at 8 dpf. The *dldh^cri3^* mutation increased mitochondrial elongation, as was potentially attributable to increased mitochondrial fusion based on observations of increased mitochondrial average area and decreased mitochondrial number detected in the mutant larval cells. Increased mitochondrial fusion may occur in response to a metabolic stress condition, as an adaptive response to protect mitochondria from degradation (35, 36). Indeed, mitochondria adjust their status and functions by modifying their shape and enabling intricate quality control. For example, mitochondria can respond to autophagy by promoting elongation (35, 36), protecting mitochondria from degradation, and promoting mitochondrial ATP production during stress conditions, such as oxidative stress (37, 38). Further studies will be important to investigate proteins and candidate therapeutic targets — such as OPA1, MFN1, and Drp1 — that are involved in mitochondrial cristae remodeling and dynamics in DLD-deficiency models.

Cristae serve as functional biochemical compartments in mitochondria where ATP is synthesized. Since cristae shape plays a critical role in the organization of OXPHOS protein clusters, their shape and condition are presumed to affect the performance of the RC enzyme complexes and supercomplexes and are assumed to be critical for metabolic adaptation (39). Interestingly, *dldh^–/–^* zebrafish mitochondrial cristae ultrastructure and density were severely altered in most of the mutant tissues analyzed, despite the enzyme activity of each RC complex being relatively unaffected. Although we note that RC activities and OXPHOS measurements were made on whole organism populations lysate, this result was still unexpected. Indeed, the OXPHOS complexes are strategically dispersed on mitochondrial cristae, where ultrastructure maintenance is required to allow for optimal OXPHOS capacity. We postulate that the observed retention of RC activity in the mutant larvae could be due to the slow turnover of RC proteins in membrane fragments or by some other unknown mechanism. Therefore, despite RC enzyme complexes remaining functional in *dldh^–/–^* zebrafish larvae, we would expect that OXPHOS impairment and impaired proton transfer would be detected at a later stage, if that were not prohibited by early animal death. Consistent with the retained function observed from RC complexes, the ATP level was not affected in *dldh^–/–^* larvae. However, in many cases of modest ATP demand, ATP levels can be maintained by increased glycolysis, which we suspect contributed to the observed high lactate level in *dldh^–/–^* zebrafish larvae. Future isotopic studies of glycolytic flux in the *dldh^cri3^* model will be important in the resolution of the relative contribution of aerobic and anaerobic respiration to maintain their ATP production under basal or stressed conditions.

Surprisingly, increased β-tubulin and VDAC protein levels were observed in *dldh^–/–^* zebrafish, and this can be correlated to changes in mitochondrial morphology. Indeed, tubulin is a fundamental component of microtubules (40), which form a network that is connected to mitochondrial positioning. Mitochondrial function strictly depends not only on mitochondrial structure and dynamics, but also on their cellular location (41, 42). Mitochondria and cytoskeleton interaction is crucial for normal mitochondrial morphology distribution and motility. Previous studies have shown that, in cardiomyopathy models, mitochondrial elongations are followed by microtubules and are involved in mitochondrial dynamics (12, 13). Furthermore, the interaction of microtubules with the outer membrane protein VDAC is directly involved in the coordination of mitochondrial function (43). Additionally VDAC is essential for the interaction between mitochondria and lysosomes, which may affect VDAC proteolysis and/or mitophagy (44). Indeed, we detected an increased level of VDAC protein in the *dldh^–/–^* zebrafish larvae, which may reflect increased mitochondrial mass that was also detected by TEM in intestinal cells.

Mitochondrial proliferation has been reported in muscle biopsies of patients with DLD deficiency (45). In the *dldh^–/–^* zebrafish tail, mitochondrial structure was mostly affected in the fast-twitch fibers, which, like the liver and intestine, showed an increased fraction of damaged mitochondria. Damaged mitochondria were characterized by fewer cristae and wider intramitochondrial intercristae spaces, decreased solidity with loss of the rounded mitochondrial shape, and loss of the outer mitochondrial membrane integrity. While mitochondrial number per cross-sectional area is unchanged in the homozygous mutants’ hepatocytes relative to controls, the number of mitochondria per cross-sectional fiber/cell area was decreased in both fast-twitch fibers and intestinal cells. Therefore, the response of mitochondrial metabolism, function, and dynamics to DLD deficiency are likely to vary depending on the specific tissue analyzed.

Interestingly, we noticed that mitochondrial ultrastructure was more severely affected by DLD deficiency in fast-twitch fibers than in slow-twitch muscle fibers. Indeed, while oxidative slow-twitch fibers have slow contraction rates, high mitochondrial content, increased reliance on OXPHOS, and high resistance to fatigue, glycolytic fast-twitch fibers produce energy by anaerobic glycolysis and have rapid contraction rates, lower mitochondrial content, decreased reliance on OXPHOS, and low resistance to fatigue. Fast-twitch fibers primarily rely on glycolysis and PDHc activity, which links glycolysis with the TCA cycle by catalyzing the conversion of pyruvate into acetyl-CoA. Since PDHc activity is compromised in *dldh^–/–^* zebrafish larvae, this may explain why mitochondrial damage was mostly detected in their fast-twitch fibers and not in slow fibers (46).

Zebrafish are growing increasingly attractive for preclinical translational modeling of quantitative aspects of neuromuscular function, swimming activity, and survival in different genetic subtypes of PMD, which can then be used to identify and optimize candidate therapies that improve their survival and/or function (47). Indeed, patients with PMD commonly have impaired movement, muscle weakness, imbalance, and impaired coordination (48). Despite neuromuscular dysfunction being the hallmark symptom in patients with PMD, few preclinical studies have been reported that quantify neuromuscular activity in translational PMD animal models.

Zebrafish larvae motor performance quantification with automated swimming activity assays performed in cyclic light-dark period exposures was established as a reliable and high-throughput phenotypic outcome for DLD deficiency candidate treatment screens. Indeed, we have recently demonstrated that combining automated, whole-organism assays of neuromuscular activity with in-depth studies of mitochondrial structure in high-energy–demand tissues of PMD animal models can serve to quantifiably assess disease severity and identify new druggable targets and/or candidate therapies to prioritize for further evaluation in PMD clinical research trials (47, 49). Here, swimming activity assays in *dldh^–/–^* zebrafish were successfully used to screen 6 (thiamine, lipoic acid, DCA, probucol, elamipretide, TAT-DLD) therapy candidates at variable exposure time courses and concentrations, with validation studies performed at whole-animal (survival, swimming activity, intermediary metabolites, mitochondrial function) and/or organ-specific tissue (liver pathology, mitochondrial ultrastructure) levels. Indeed, these studies reveal that significantly improved swimming activity was achieved in 7 dpf *dldh^–/–^* zebrafish larvae following treatment with either probucol (5 μM) or thiamine (0.1 mM or 0.5 mM). However, mechanistic studies investigating probucol’s effect failed to show a corresponding rescue of biochemical metabolite alterations or of liver pathology. Surprisingly, we observed that thiamine had a narrow therapeutic index in the *dldh^–/–^* model, where 5 mM thiamine completely ablated their swimming activity. We postulate that the absence of the E_3_ subunit may expose the noncovalently bound thiamine cofactor of the E_1_ subunit to exchange with excess thiamine and enhance the production of ROS (25, 30). Conversely, we observed improved pyruvate metabolism with 25 mM DCA treatment and improved DLD expression following TAT-DLD protein injection but no corresponding improvement with either treatment at the level of *dldh^–/–^* larval swimming activity. We postulate that the lack of swimming improvement upon DCA exposure reflects the broader biochemical pathology of E_3_ enzyme dysfunction, beyond isolated PDHc involvement. Furthermore, it remains possible that failure of TAT-DLD to rescue the impaired swim activity reflects an inability to provide sufficiently high treatment levels due to microinjection volume limits. Collectively, these data provide objective evidence for a therapeutic benefit of low-dose thiamine, which is often empirically prescribed in PDHc-deficiency disorders including DLD deficiency, as well as probucol (14, 21). Furthermore, these studies provide positive treatment controls (probucol, thiamine) that can be used for future high-throughput drug and/or genetic library (21) screens to potentially identify more potent therapies for DLD deficiency that have favorable safety and efficacy profiles.

In summary, to our knowledge, this preclinical study establishes *dldh^cri3^* zebrafish as the first viable vertebrate animal model of human DLD-deficiency disease. *dldh^–/–^* larvae exhibit the full spectrum of intermediary metabolic deficits anticipated to be caused by Dldh deficiency, extending beyond PDHc deficiency to include metabolite evidence of impaired enzymatic activity in all mitochondrial α-keto acid dehydrogenases but not in the GCS. This complex metabolite profile is amenable to future investigations of mechanistic targets and candidate drug screening in DLD deficiency. In-depth ultrastructural analyses of mitochondria in the *dldh^–/–^* zebrafish provided an intriguing connection between intermediary metabolic disruption and tissue-specific mitochondrial ultrastructural responses in DLD deficiency, which hold potential to corroborate beneficial cellular effects of drug leads identified in high-throughput candidate treatment screens of phenotypic outcomes. Furthermore, preclinical evaluation of candidate treatments provided objective evidence of improved swim activity in *dldh^–/–^* zebrafish treated with either low-dose thiamine (0.5 mM and 1 mM) or probucol (5 μM), and it is amenable to future high-throughput library screens to enable identification and prioritization of additional treatment candidates to support their evaluation in clinical trials that evaluate therapeutic benefits in patients with DLD disease.

## Methods

### Sex as a biological variable

Sex is indeterminate in zebrafish juvenile hermaphrodite larvae at 7 dpf, and therefore was not considered as a biological variable (50, 51).

### D. rerio strains and maintenance

WT zebrafish were obtained from the zebrafish international research center, ZIRC (https://zebrafish.org/). The *dldh^cri3^* mutant line was created in CHOP Zebrafish Core using CRISPR/Cas9 gene editing with a sgRNA targeting a sequence next to a known disease-causing mutation (human E375K). AB WT embryos were injected at the 1-cell stage and allowed to grow to adulthood to generate the F0 generation. F0 offspring were analyzed by PCR and DNA sequencing to establish the stable mutant line, *dldh^cri3^*, harboring a 5 bp deletion, resulting in a stop codon at the site of the mutation (c.1180_1184delGTGTG, p. C371X [NDM_201506.1]; sequence location shown in Supplemental Figure 1). Subsequent outcrossing of *dldh^+/–^* zebrafish to AB WT zebrafish provided F2 and F3 generations. The *dldh^cri3^* mutation results in a restriction fragment length polymorphism (RFLP) by removing an MslI restriction site (R0571S, New England BioLabs). The genomic region was amplified using primers: 5′-TTCCTCTTTAAGCTGTTCCTCA-3′, 5′-GTCCGGTCCGTAACTGAAAA–3′. The size of the DNA fragments was analyzed on an agarose gel after digestion with MslI. Adult *dldh^+/–^* zebrafish were mated pairwise to obtain *dldh^–/–^* homozygous mutants, and the resulting embryos were collected and sorted at 0 dpf. Embryos were sanitized with sodium hypochlorite (0.003%) and treated with Pronase by standard methods (52) to promote uniform hatching at 1 dpf; at 2 dpf, Pronase was removed. Embryos were grown in embryo media (E3; 5.0 mM NaCl, 0.17 mM KCl, 0.33 mM CaCl_2_, 0.33 mM MgSO_4_). *dldh^–/–^* larvae were identified by lack of an inflated swim bladder by 5 dpf. Homozygosity was verified by RFLP genotyping. Zebrafish maintenance and husbandry were performed at 28°C in the CHOP Zebrafish Core.

### Immunoblotting analysis

Thirty zebrafish larvae were suspended in 200 μL of RIPA buffer supplemented with protease inhibitor cocktail (Sigma-Aldrich). Larvae were lysed on ice using an IKA RW 20 Digital Mixer (IKA Works) and centrifuged for 20 minutes at 213,000*g*. Supernatant was collected, and the protein concentration was determined using a Pierce BCA protein assay kit (Thermo Fisher Scientific). Equal amounts (30 μg) of whole lysate proteins were loaded onto 4%–15% Tris-glycine SDS gels, electrophoresed and wet transferred to nitrocellulose membranes, blocked in blocking buffer (Intercept TBS Blocking Buffer, LI-COR Biosciences) for 1 hour, and incubated with specific primary antibodies in blocking buffer for 1 hour at room temperature or overnight at 4°C. The membranes were further incubated with secondary antibodies (IRDye conjugated secondary antibodies: 800CW goat anti–rabbit IgG and 680RD goat anti–mouse IgG, LI-COR Biosciences) in blocking buffer containing 0.2% Tween20 for 1 hour at room temperature and visualized using an Odyssey CLx Infrared Imaging System. The relative protein band densities were quantified using ImageJ (NIH) (53, 54). The following antibodies were used: anti-DLD (1:1,000 dilution, Abcam, ab133551), anti-COXIV (1:1,000, Cell Signaling Technology, 4850), anti-CS (1: 1,000, Abcam, ab96600), anti- VDAC (1:1,000, Cell Signaling Technology, 4661), anti–β-tubulin (1:5,000, MilliporeSigma, 05-661), and anti–β-actin (1:5,000, MilliporeSigma, A1978). Three independent biological replicate experiments were completed per condition.

### Survival analysis of dldh–/– zebrafish larvae

To monitor survival beyond 1 week in Petri dishes, zebrafish larvae were fed paramecium twice daily starting at 5 dpf. Viability was scored by the presence or absence of a heartbeat. Scoring was performed twice daily until all *dldh^–/–^* larvae were scored as inviable, after which all surviving fish were euthanized by immersion in ice water. Statistical analysis was performed using the log-rank (Mantel-Cox) test with GraphPad Prism (GraphPad Software). Analysis was performed on 300 larvae each fish line (*dldh^+/+^, dldh ^+/–^*, and *dldh^–/–^*) across 3 biological replicates. 

### Neuromuscular swimming activity analysis in zebrafish larvae

Swimming activity was assessed by monitoring larval zebrafish movement before and after transition between light and dark periods at 7 dpf using the ZebraBox tracking system and ZebraLab software (ViewPoint Life Sciences). Larvae were placed individually into wells of a 96-well plate with Tris-buffered embryo E3 media and allowed to acclimate under light (100% light power) in the ZebraBox system for 20 minutes prior to experimentation. The experiment consisted of 4 cycles of a 10-minute dark period (0% intensity) followed by a 10-minute light-on period (100% intensity, 1,880 lux). Technical replicates each included at least 12 control larvae and 12 *dldh^–/–^* larvae. Three biological replicate experiments were performed per condition. AUC analysis was performed using GraphPad Prism to analyze larval zebrafish activity during the dark cycles.

### Zebrafish larvae liver size analysis

A subset of 14 *dldh^–/–^* and 11 combined WT sibling *dldh^+/+^* and *dldh^+/–^* 7 dpf larvae were anesthetized with 4 mg/mL tricaine in E3, buffered with Tris (pH 7.2), and imaged using a dissecting microscope (Olympus MVX10), an Olympus DP73 camera, and Olympus CellSens imaging software. Zebrafish liver area was measured using ImageJ software (53, 54). Screening of candidate drug preliminary efficacy to normalize liver size was also evaluated in *dldh^–/–^* zebrafish larvae at 6 dpf and 7 dpf after incubation for 48 or 72 hours.

#### Zebrafish larvae liver fat analysis by Oil Red O staining.

Whole-mount Oil Red O staining of control and *dldh*^–/–^ larvae at 8 dpf was performed, as previously described (55). In brief, 10 larvae of each genotype were fixed in 4% paraformaldehyde overnight at 4°C before being washed 3 times in PBS and 0.5% Tween (PBST). Larvae were subsequently incubated in 0.3% Oil Red O dissolved in 100% isopropyl alcohol for 15 minutes. After removing the isopropyl alcohol, larvae were rinsed quickly in PBST and incubated in 4% paraformaldehyde for 10 minutes. The paraformaldehyde solution was removed, and larvae were stored in 50% glycerol in PBST. Images were taken using a dissecting microscope (Olympus MVX10), an Olympus DP73 camera, and the CellSens imaging software, with qualitative analysis performed on at least 10 larvae per condition across 3 biological replicates.

### Zebrafish larvae electron microscopy analyses

Seven dpf *dldh*^–/–^ and combined WT sibling *dldh^+/+^* and *dldh^+/–^* zebrafish larvae were collected and fixed in 6% glutaraldehyde and 1% formaldehyde (vol/vol) in 0.1M cacodylate buffer (pH 7.4) at room temperature and were processed for TEM; ultrathin sections were made, analyzed, and stored at 4°C (49). Cross sections were obtained to perform quantitative analysis of mitochondrial ultrastructure in hepatocytes, intestinal cells, and tail skeletal muscle fast-twitch fibers. Three mutant and WT larvae were analyzed, and measurements were obtained using ImageJ.

### Zebrafish larvae GC/MS metabolite analyses

Extracted metabolites from zebrafish larvae population samples were converted to their *N*-methoxyoximes and silylated to their trimethylsilyl derivatives by standard methods (56). Briefly, each sample consisted of 100 pooled flash-frozen 7 dpf larvae. Larvae were homogenized by grinding with an electronic pestle in 150 μL of ice-cold 4% perchloric acid (PCA) and subsequently frozen in liquid nitrogen. Each sample was then thawed and sonicated on ice at 10% amplitude for 4 seconds with alternating 0.5 seconds on/off periods and centrifuged at 25,000*g* at 4°C for 5 minutes to precipitate the protein. After transferring the supernatant to a new tube, the protein was quantified using the Pierce BCA protein assay kit (Thermo Fisher Scientific, 23225). Potassium perchloroacetate was precipitated by the addition of ~9.5 μL of 2N potassium carbonate and centrifuged at 25,000*g* for 5 minutes. The supernatant was transferred to a new tube, and 50 μL of 300 mg/mL methoxyamine hydrochloride was added. In total, 10 μL of 1 μg/μL undecanedioic acid in 0.1N NaOH was added as an internal standard. The pH was brought to 10–11 with ~80 μL of 2N NaOH, and the samples were derivatized at 70°C for 30 minutes. After cooling, ~50 μL of 5N HCl was added to pH 1–2, and the volume was adjusted to 1 mL with water. The sample was transferred to a glass tube filled with 1 g of NaCl and vortexed. After addition of 1 mL of ethanol/ethyl acetate (20:80; v:v) and vortexing for 30 seconds, the sample was centrifuged at 1,000*g* for an additional 5 minutes, and the organic layer was transferred to a new glass tube. The water layer was extracted twice with ethanol/ethyl acetate (20:80; v:v). The pooled organic extracts were dried under a stream of nitrogen and resuspended in 1 mL methanol/dichloromethane (1:1; v:v), dried under N_2_, silylated by the addition of 50 μL of ethyl acetate and 50 μL *N,O*-bis(trimethylsilyl)trifluoroacetamide + 1% TMCS silylation reagent (Thermo Fisher Scientific, 38832), and reacted at 70°C for 30 minutes. Following derivatization, the sample was centrifuged at 4,000*g* for 5 minutes, and the supernatant was transferred to an autosampler vial.

Each sample (1 μL) was injected into an Agilent 7890A GC/MS system fitted with a 5975C inert XL mass selective detector with a Triple-Axis Detector (Agilent). The GC was maintained at 70°C for 2 minutes followed by a ramp of 5°C/min to 250°C and then 10°C/min to 300°C with a hold for 10 minutes. Full-scan MS data were acquired over the *m/z* range of 50–550. Positive peak identification was based on comparison with authentic standards and from the Agilent GC/MS 3 Data Analyzer Mass Spectrometry Software. Each metabolite is reported as μg relative to the internal standard of undecanedioic acid assuming an equal detector response and was normalized to protein in the pooled sample. Statistical analysis used Welch’s unpaired *t* test in GraphPad Prism (*n* = 6 for each condition).

### Fish larvae sample preparation for biochemical assays

For biochemical assays, 20 embryos were collected per tube and washed twice with E3 buffer. After buffer removal, embryos were immediately frozen in liquid N_2_ and stored at −80°C. For respiratory chain (RC) complex and CS assays, frozen zebrafish larvae were homogenized in a mitochondrial isolation buffer (250 mM sucrose, 20 mM Tris-HCl, 3 mM EDTA [pH 7.4]; Sigma-Aldrich) on ice with a motorized pestle, followed by 3 freeze/thaw cycles with liquid nitrogen. The lysate was subjected to differential centrifugation (750*g* for 5 minutes followed by 10,000*g* for 10 minutes) to obtain a mitochondrial-enriched fraction.

For ATP and lactate assays, frozen 7 dpf zebrafish larvae were homogenized in ice-cold 0.5M PCA by grinding with a motorized pestle followed by 2 seconds of sonication and 1 freeze/thaw cycle in liquid nitrogen/room-temperature water. Supernatant was centrifuged 15 minutes at 16,000*g* and subsequently neutralized by ice-cold 1M potassium carbonate.

### Zebrafish larvae DLD E3 and RC enzyme activities

For enzyme activity assays, zebrafish larval mitochondria-enriched larval fractions were used in spectrophotometric assays performed at 30°C in 170 μL final volume using a Tecan Infinite 200 PRO plate reader (Tecan Trading). DLD E_3_ activity was determined by the reduction of 2,6 dichlorobenzenone-indophenol (2,6-DCPIP) at 600 nm (ε_600_ = 21 mM^−1^ cm^−1^) (6). DLD assay buffer contained 50 mM KH_2_PO_4_ (pH 7.0), 1.25 mM EDTA, 400 μM lipoamide, and mitochondria-enriched zebrafish extract; the reaction was started with 100 μM NADH. Rates were calculated after subtraction of lipoamide-insensitive activities. The specific activity was normalized following Bradford assay of the protein concentration (57).

Complex I and complex II enzyme activities were determined by the reduction of 2,6-DCPIP (47) in assay buffer (25 mM KH_2_PO_4_ [pH 7.4], 5 mM MgCl_2_, 3 mg/mL BSA, 25 μM ubiquinone-Q1, 5 μM antimycin-A, and mitochondria-enriched larval zebrafish extract). Reactions were initiated by addition of 150 μM NADH in the presence and absence of 5 μM rotenone; complex I rates were calculated after subtraction of the rotenone-insensitive activity. The assay for complex II activity additionally contained 5 μM rotenone to inhibit complex I. Reactions were started by addition of 20 mM succinate. Complex IV enzyme activity was measured by following the oxidation of reduced cytochrome *c* at 550 nm (ε_550_ = 21 mM^−1^ cm^−1^) in 5 mM KH_2_PO_4_ (pH 7.4), 5 mM MgCl_2_, 0.015% n-dodecyl-β-d-maltoside, 5 μM antimycin-A, 5 μM rotenone, and mitochondria-enriched zebrafish extract. Reactions were initiated with 15 μM reduced cytochrome *c*. Rate constants were obtained from a fit to a first order reaction. Specific activities were calculated by obtaining the protein concentration by Bradford assay (57).

Zebrafish larvae polarographic analysis of mitochondrial O_2_ flux (*J*_O2_). *J*_O2_ under different conditions was recorded following the sequential addition of substrates and inhibitors to determine the integrated and individual functions of the individual RC complexes using an Oxygraph-2k (Oroboros Instruments) (58) and following published terminology (59). Details are in Supplemental Figure 3. GraphPad Prism was used for statistical analysis by Welch’s unpaired *t* test (*n* = 3 for each condition).

### Zebrafish larvae lactate and pyruvate assays

Lactate oxidase (LOX) and pyruvate oxidase (POX) colorimetric end point assays were used to quantify lactate and pyruvate (47). LOX and POX produce H_2_O_2_ by the oxidation of lactate and pyruvate, respectively. *N*-(carboxymethylaminocarbonyl)-4,4′-bis(dimethylamino)diphenylamine sodium salt (DA-64, BOC Sciences, 115871-19-7) is readily oxidized by H_2_O_2_ (60). Mitochondria-enriched extract was added to 155 μL of assay mixture (0.2 mM DA-64, 1 mM EDTA, 0.1% Triton X-100, and 5 U/mL horseradish peroxidase [HRP] in 100 mM HEPES [pH 7.4]; POX additionally requires 10 μM FAD, 0.2 mM TPP, and 10 mM MgCl_2_), mixed thoroughly and then incubated at 37°C for 3 minutes; the reaction was initiated with 0.02 U of freshly prepared enzyme, and the 727 nm absorbance was monitored for 15 minutes. Lactate and pyruvate concentrations were calculated using lactate and pyruvate standard curves.

### Zebrafish larvae ATP and NAD+ quantitation by HPLC

Separation of ATP and NAD^+^ from zebrafish larvae homogenates was performed as described (6).

### Zebrafish larvae candidate therapy screen

Preliminary drug screening was conducted using the following DLD deficiency treatment candidates: thiamine (in H_2_O), lipoic acid (in EtOH), DCA (in H_2_O and obtained from MilliporeSigma or Saol Therapeutics), elamipretide (SBT0259 and SBT0272, in DMSO and obtained from Stealth Therapeutics), and probucol (in DMSO). Concentrations, treatment incubation times, and screening assays performed are in Table 1. Swimming activity assays at 7 dpf larval stage were performed in ≥ 3 biological replicates with *n* = 12 animals per biological replicate for each condition and zebrafish line.

Following preliminary screens, thiamine (0.1, 0.5, 1.0, and 5.0 mM) and probucol at 5 μM were selected for replicate swim activity and/or liver and biochemical analyses. Specifically, WT and *dldh^–/–^* larvae were incubated with thiamine or 5 μM probucol diluted in < 0.01% DMSO from 4 dpf to 6 dpf, and swim activities were recorded and analyzed at 7 dpf in at least 3 biological replicates. One-way ANOVA was applied to the thiamine data, while the probucol data were analyzed with Student’s *t* test. Liver size and Oil Red O analyses were performed for a subset of at least 3 fish per condition, and metabolomic analyses of control and drug-treated animals were performed by GC/MS.

In addition, while DCA treatment from 5 to 7 dpf was not found to improve *dldh^–/–^* larvae swim activity at 7 dpf, additional study was performed supported by Saol Therapeutics to evaluate whether prolonged treatment from earlier in life would improve outcomes. Swim activity and biochemical metabolite levels of lactate and pyruvate were evaluated following prolonged DCA treatments from 0 dpf (at 5 mM and 25 mM) in 7 dpf *dldh^–/–^* larvae. DCA was refreshed on 1 dpf and 5 dpf. For lifespan analysis, DCA was additionally refreshed daily starting on 7 dpf until no surviving zebrafish remained. Three biological replicates were performed per condition, with analyses completed as described above.

### Zebrafish larvae TAT-DLD fusion protein studies

The development of an enzyme-replacement therapy to treat DLD deficiency relies on the use of a recombinant form of human DLD expressed in and purified from *E*. *coli*. Two different TAT-DLD fusion proteins were tested, named “1” and “2”. TAT-DLD–protein-1 was produced by overexpression in *E. coli* of the plasmid pD451-SR that incorporated the DLD sequence preceded by the 11 aa cell penetrating peptide from the HIV-TAT protein (61) and the 34 aa mitochondrial targeting sequence (ATUM). The 30 L *E. coli* fermentation and induction with 1 mM IPTG was conducted at the University of Iowa Center for Biocatalysis and Bioprocessing (CBB; Iowa City, Iowa, USA). Following lysis using a Microfluidizer at 17,000 psi and centrifugation of the lysate at 15,000*g* for 40 minutes, purification of the TAT-DLD–protein-1 was accomplished by SP-Sepharose FF and Q-Sepharose FF chromatography (Cytiva). Purity was monitored by SDS-PAGE. TAT-DLD–protein-2 was synthesized at the Department of Biochemistry and Molecular Biology, Institute for Medical Research-Israel-Canada, The Hebrew University-Hadassah Medical School (Jerusalem), as previously described (5, 8). TAT-DLD protein was injected into the yolk of freshly fertilized WT and *dldh^–/–^* embryos (1 dpf) and again at 4 dpf. TAT-DLD protein, 0.237 ng in 900 pL, was injected into each embryo along with FITC-Dextran to control for successful injection and retention of the injected liquid (5, 8). The efficacy of TAT-DLD enzyme-replacement therapy was tested by monitoring larvae for survival and swim activity using the ZebraBox platform and immunoblot analysis (anti-DLD, 1:1,000 dilution, Abcam, ab133551) of DLD expression on isolated protein from 7 dpf larvae.

### Statistics

GraphPad Prism was used for statistical analyses, using 2-tailed Student’s unpaired *t* test unless otherwise noted. Three or 4 biological replicates were performed for each condition. Significance level was determined as *P* < 0.05.

### Study approval

All protocols and methods were approved by the CHOP IACUC (protocol no. 21-001154) and follow the regulations for care and use of *D*. *rerio*.

### Data availability

Digital data for the statistical analyses of all graphs are available in Supporting Data Values.

## Author contributions

MJF conceived of and designed the study, obtained study funding, and assisted in manuscript preparation. ML performed sample preparation for EM analysis, analyzed EM ultrastructural data, performed swimming activity experiments, and reviewed all study data and figures. ENO performed biochemical analyses in zebrafish larvae. DI performed zebrafish husbandry and sample collection, HPLC and GC/MS metabolite profiling assays, Oxygraph-2k oximetry experiments, and swim activity analyses of DCA and probucol. CR performed immunoblotting experiments and analysis. CS designed and performed CRISPR/Cas9 gene editing in zebrafish to create the DLD deficiency zebrafish model. CB and PK performed zebrafish survival assays, genotyping, TAT-DLD fusion protein injections, drug testing, and swimming activity assays. RX advised and assisted with all statistical analyses. NDM assisted with zebrafish swimming activity data analysis and performed the protein homology alignment. ML, DI, VEA, and MJF drafted the manuscript. All authors reviewed and approved the final manuscript.

## Supplementary Material

Supplemental data

Unedited blot and gel images

Supporting data values

## Figures and Tables

**Table 1:**
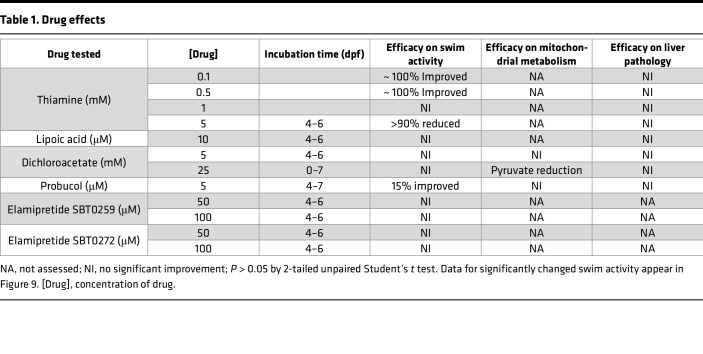
Drug effects

## Version 1 08/20/2024

In-Press Preview

## Version 2 09/24/2024

Electronic publication
