# Supplementary material for: dldhcri3 zebrafish exhibit altered mitochondrial ultrastructure, morphology, and dysfunction partially rescued by probucol or thiamine
Source: JCI Insight. 2024 Aug 20;9(18):e178973. doi: 10.1172/jci.insight.178973 (PMC11457866; doi:10.1172/jci.insight.178973)
Supplement: Supplemental data [file jciinsight-9-178973-s157.pdf]

SUPPLEMENTAL MATERIAL

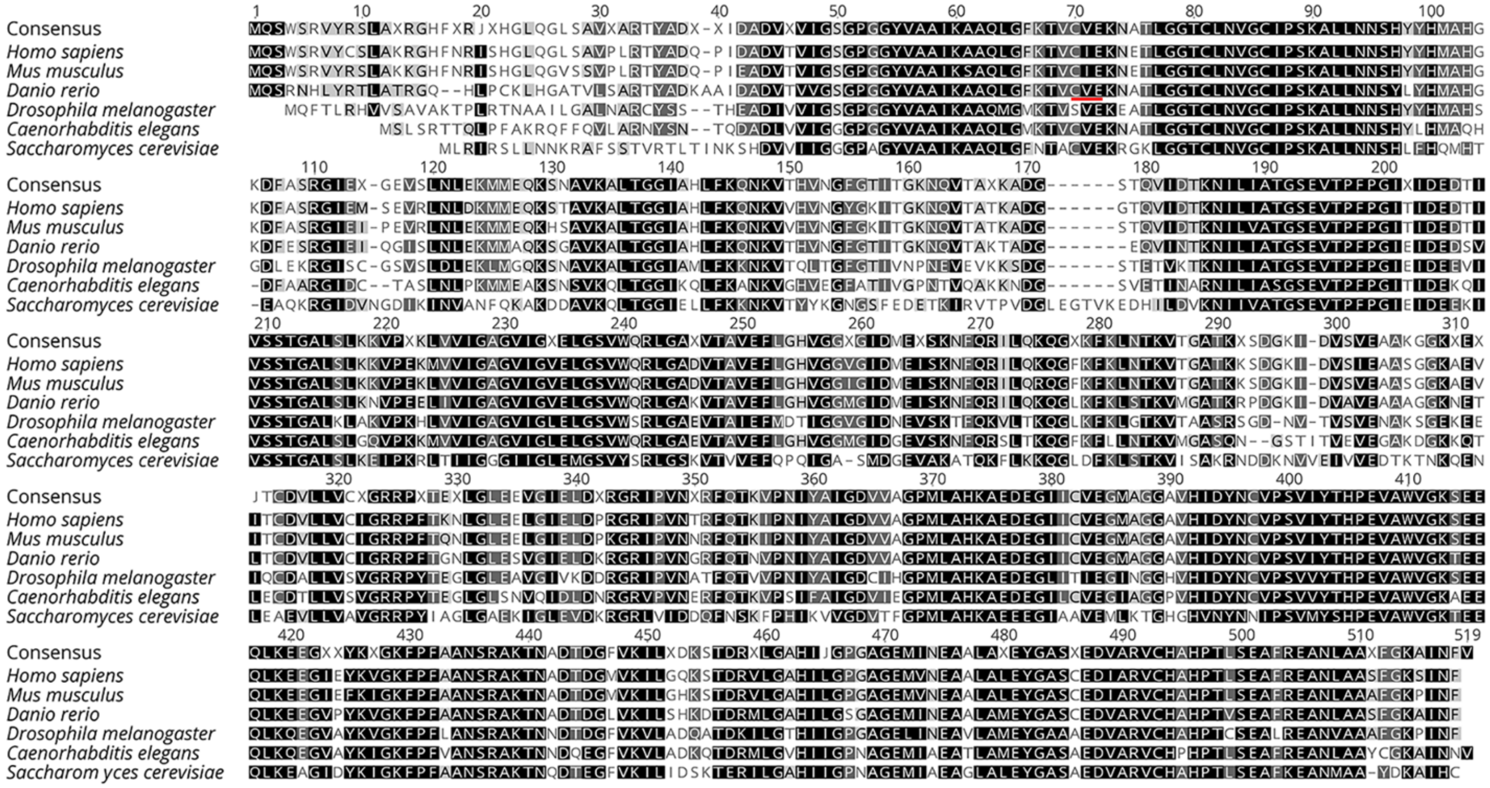

Supplemental Figure S1. Protein alignment of the DLD in *Danio rerio*, *Homo sapiens* and other species. Alignment generated with ClustalW version 2.0.12. Identities are identified with a black background. The mutant zebrafish carry a 5 bp deletion, TCC-AC, which causes a deletion of CVE and a frame shift mutation (underlined in red).

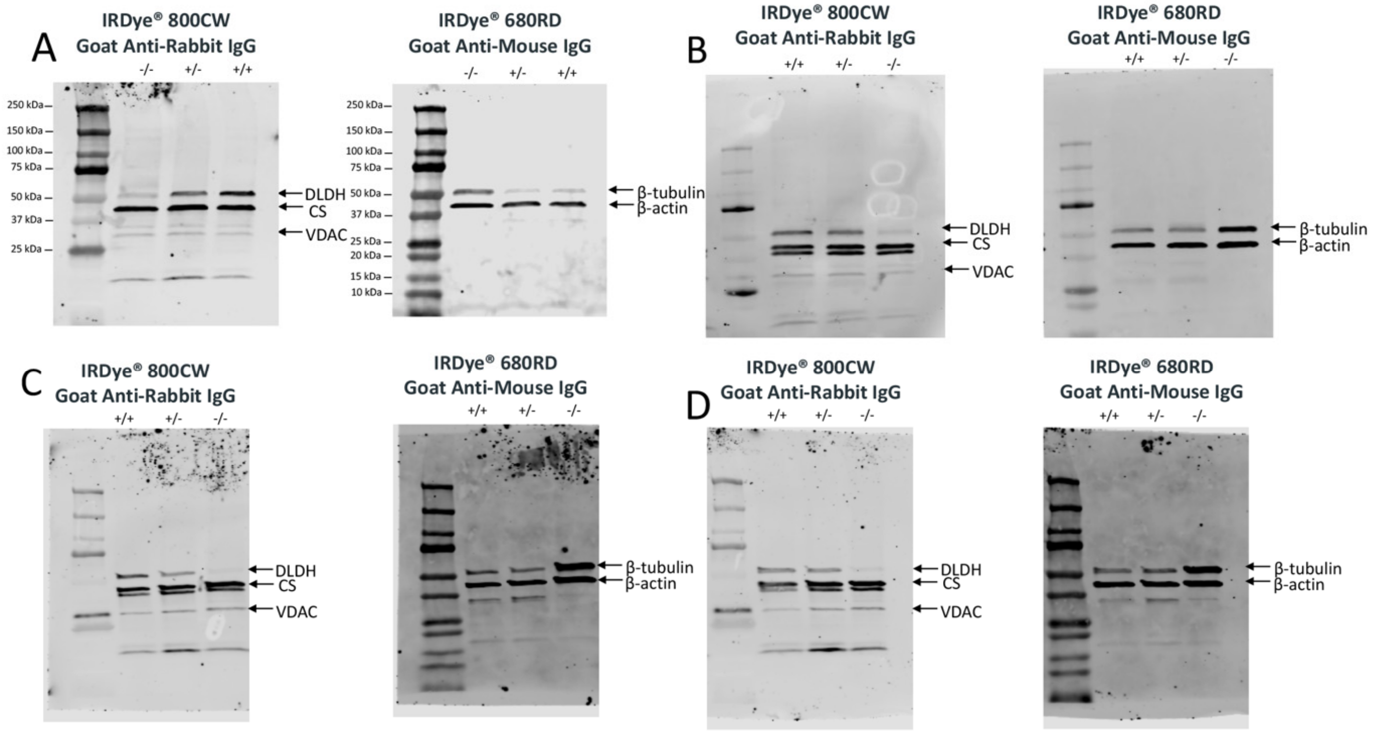

Supplemental Figure S2. (A-D) Original Western immunoblot images used for Figure 1D. Indicated protein levels in *dldh*$^{+/+}$, *dldh*$^{+/-}$ and *dldh*$^{-/-}$ 7dpf zebrafish larvae were analyzed in four biological replicate experiments. β-actin was used as a loading control.

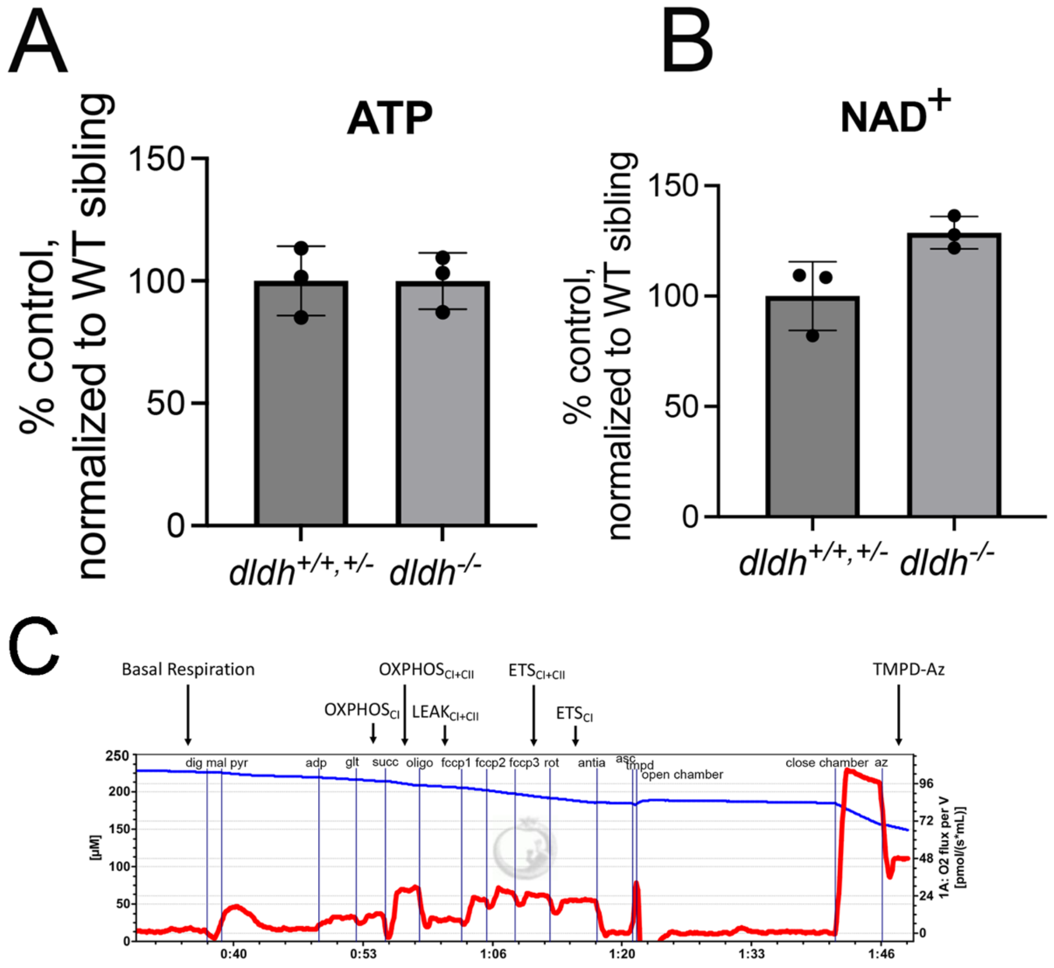

Supplemental Figure S3. Biochemical analysis of ATP and NAD^+ levels in 7 dpf *$dldh^{-/-}$* larvae and polarographic and spectrophotometric analyses of mitochondrial respiratory chain function in *DLD* deficient zebrafish larvae. A) ATP and NAD^+ levels were not significantly changed in *$dldh^{-/-}$* larvae relative to sibling WT and heterozygous larvae at 7 dpf. **B)** Oxygen flux (O_2 flux J_{O2}) measurements normalized to mitochondrial protein were made by high-resolution polarography using an Oxygraph-2k (Oroboros) in 5 dpf *$dldh^{-/-}$* zebrafish larvae homogenate samples relative to WT controls. The above is an example trace of the oxygen concentration (blue line, left y-axis) and flux (red line, right y-axis). Each vertical blue line depicts when either a substrate, uncoupler, or inhibitor was added. Measurements were taken as an average once the flux stabilized. FCCP was titrated in until the flux did not further increase, and the measurement is at the maximal respiration. After addition of TMPD, the chamber was opened for 20 minutes and closed again before the measurement was taken. Black arrows depict where the measurements were taken in **Figure 3 C**. Dig, digitonin; mal, malate; pyr, pyruvate; adp, adenosine diphosphate; glt, glutamate; FCCP, carbonyl cyanide-p-trifluoromethoxyphenylhydrazone; rot, rotenone; antia, antimycin a; asc/tmpd, ascorbate N,N,N′,N′-tetramethyl-p-phenylenediamine; az, azide. Black arrows depict where the indicated J_{O2} measurements were recorded: $OXPHOS_{CI}$, coupled OXPHOS through complex I; $OXPHOS_{CI+CII}$ coupled OXPHOS through CI and CII; $LEAK_{CI+CII}$ is the J_{O2} when ATP synthesis is completely inhibited in the presence of CI and CII substrates; ETS_{CI+CII} is the J_{O2} uncoupled from ATP synthesis in the presence of CI and CII substrates; ETS_{CII} is the J_{O2} with CI inhibited by rotenone; TMPD-az is the difference in J_{O2} determined in the presence of TMPD and after azide addition, reflecting CIV activity.

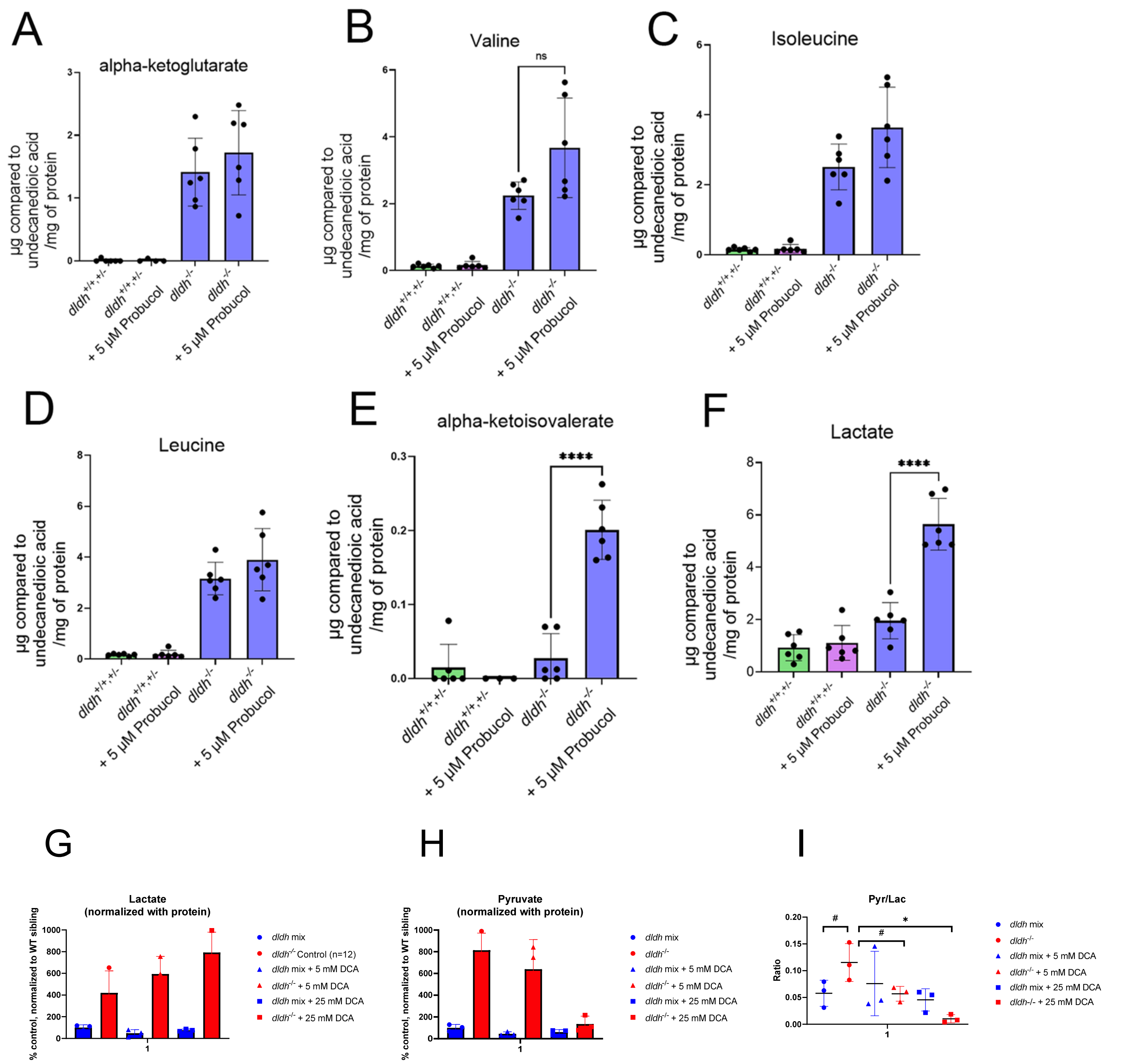

Supplemental Figure S4. 5 µM probucol treatment did not improve DLD E_3 function. **(A-F)** DLD-dependent enzymes' precursor metabolites accumulation in 7 dpf *dldh*$^{-/-}$ larvae were not improved after 5 µM probucol treatment from 5 dpf to 7 dpf. Zebrafish tissue lactate, as a proxy for pyruvate, as well as alpha-ketoisovalerate in *dldh*$^{-/-}$ larvae were significantly increased by 5 µM probucol treatment. Analyte quantitation was performed by GC/MS. n= ≥3 samples per condition, 3 biological replicates. Statistical significance was evaluated by Welch's test, where ****$p<0.0001$. ns, no significant difference seen. Each circle conveys a single replicate of 100 larvae/sample. **(G-I)** Lactate and pyruvate measured in n=20 7 dpf larvae normalized to protein concentration and to control (mixture of *dldh*$^{+/+}$ and *dldh*$^{+/-}$) zebrafish. Treatment with 5 and 25 mM DCA resulted in a dose dependent increased lactate and reduced pyruvate in three biological replicates. *$p < 0.05$.

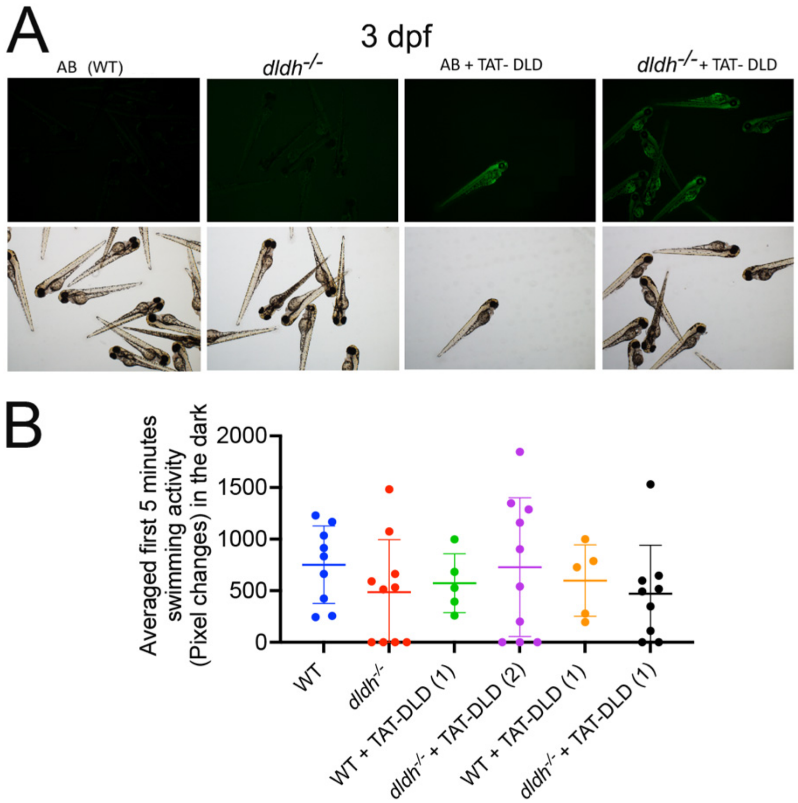

Supplemental Figure S5. TAT-DLD injections did not rescue the disease phenotype in *dldh*$^{/}$ zebrafish larvae at the level of survival or swim activity. TAT-DLD protein replacement effects were studied by injection in *dldh*$^{-/-}$ larvae. +TAT-DLD (1) = injected with the TAT-DLD protein fusion 1 (synthetized at Iowa University); +TAT-DLD (2) = injected with the TAT-DLD protein fusion 2 (synthetized at the University of The Hebrew University-Hadassah Medical School, Jerusalem, Israel). **(A) Images of embryos** injected with FITC-dextran TAT-DLD protein (green fluorescence, representative image of TAT-DLD (1) protein injected) and AB (WT) and uninjected *dldh*$^{-/-}$ zebrafish embryos at 3 dpf. Mutant were injected with TAT-DLD1, injected at day 1 post fertilization and analyzed at 3 dpf.**(B) Swimming activity** analysis of TAT-DLD injected mutant zebrafish larvae. Representative swimming activi ty of WT and mutant zebrafish larvae at 6 dpf, injected with TAT-DLD (1,2) protein showing no difference as compared to the uninjected larvae. Fish were injected with the protein at 1 and 4 dpf; swimming activity performed using the zebrabox at 6 dpf; the protocol consisted in recording the swimming activity under the following condition: 10 minutes under 100% light for acclimation, 4x 10 min 100 % light cycle, and 0% light (dark). Analysis was conducted using the averaged activity of the first 5 mins of the last three dark cycles, as represented in the graph. Three replicates were performed, but improvement was not detected.

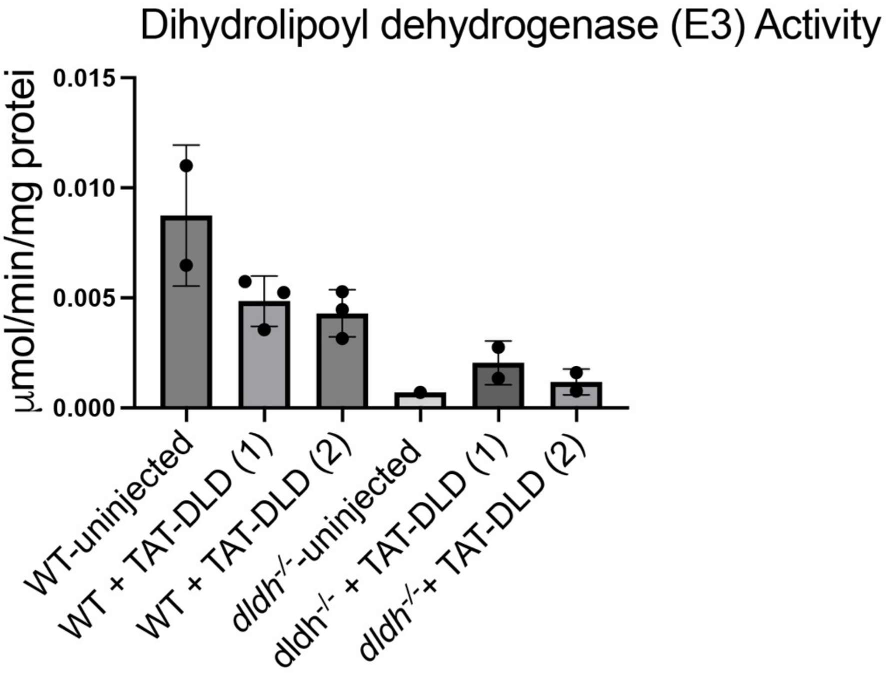

Supplemental Figure S6. DLD (E_3) activity in TAT-DLD injected *dldh*$^{-/-}$ larvae. E_3 activity was decreased in uninjected *dldh*$^{-/-}$ larvae relative to WT larvae uninjected controls and WT larvae injected with TAT-DLD protein. +TAT-DLD (1) = injected with the TAT-DLD protein fusion 1 (synthesized at Iowa University); +TAT-DLD (2) = injected with the TAT-DLD protein fusion 2 (synthesized at the University of The Hebrew University-Hadassah Medical School, Jerusalem, Israel). Larval injections with TAT-DLD protein at 1 and 4 dpf, did not rescue TAT-DLD protein levels in injected larvae quantified at 7 dpf. Each circle represents a single experiment of n= 5-10 larvae/sample.
