# Supplementary material for: dldhcri3 zebrafish exhibit altered mitochondrial ultrastructure, morphology, and dysfunction partially rescued by probucol or thiamine
Source: JCI Insight. 2024 Aug 20;9(18):e178973. doi: 10.1172/jci.insight.178973 (PMC11457866; doi:10.1172/jci.insight.178973)

Full Unedited Images for Figure 1D

Primary antibodies are as marked. In Figure 1 D the rows are mirror images so that the WT (+/+) is on the left and the KO mutant (-/-) is on the right. Note these are panels A and B of Supplemental Figure S2.

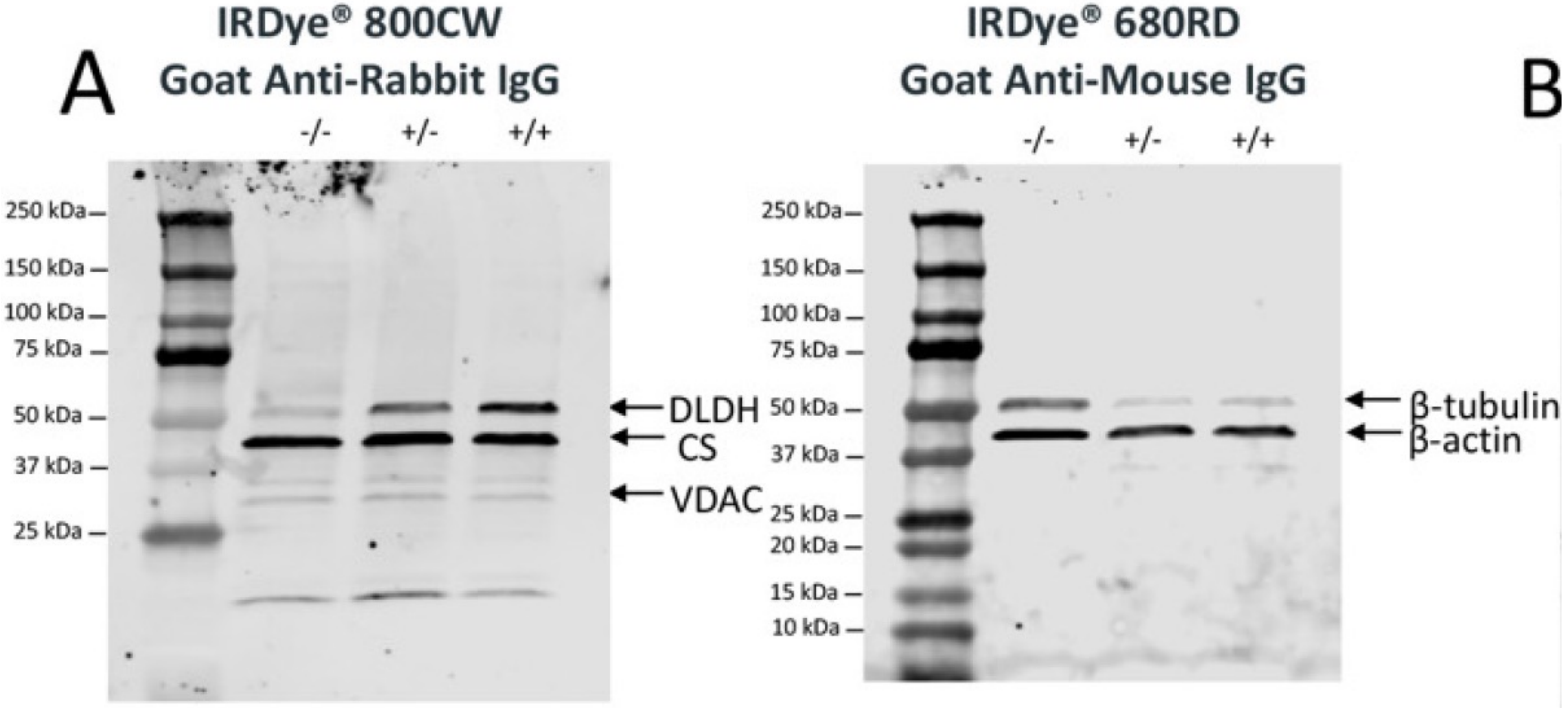

Supplement: Unedited blot and gel images [file jciinsight-9-178973-s158.pdf]
